# Exploring the potential of polymers: advancements in oral nanocarrier technology

**DOI:** 10.3762/bjnano.16.122

**Published:** 2025-10-10

**Authors:** Rousilândia de Araujo Silva, Igor Eduardo Silva Arruda, Luise Lopes Chaves, Mônica Felts de La Roca Soares, Jose Lamartine Soares Sobrinho

**Affiliations:** 1 National Institute of Science and Technology of the Health Economic-Industrial Complex - iCEIS. Federal University of Pernambuco, 50740-520, Recife, Pernambuco, Brazil

**Keywords:** drug delivery, nanoparticle, oral administration, polymer, polymeric nanoparticle

## Abstract

Polymers play a pivotal role in various drug delivery systems due to their versatility, with polymeric nanoparticles showing significant potential to overcome physiological barriers associated with oral administration. This review examines the current advancements in the application of polymers as oral nanocarriers, emphasizing key natural and synthetic polymers that enhance stability, bioavailability, and release. The physicochemical properties, biodegradability, and chemical modifications of these polymers, which promote mucoadhesion and epithelial permeability, critical factors for effective oral drug delivery, are discussed in detail. Furthermore, nanoparticle synthesis methods that enable controlled release profiles, optimized biodistribution, and improved therapeutic efficacy are also explored. Thus, polymers represent a dynamic platform for developing diverse nanocarriers for oral applications, and this review provides a valuable theoretical foundation for understanding the strategies currently employed in this field.

## Review

### 1 Introduction

The oral route is the simplest and most effective method for administering medications with minimal side effects. It is cost-effective, corresponding medications are easy to produce on a large scale, and it promotes high patient adherence to therapy. This makes this route the largest market for pharmaceutical products intended for human use [1].

**Figure 1:**
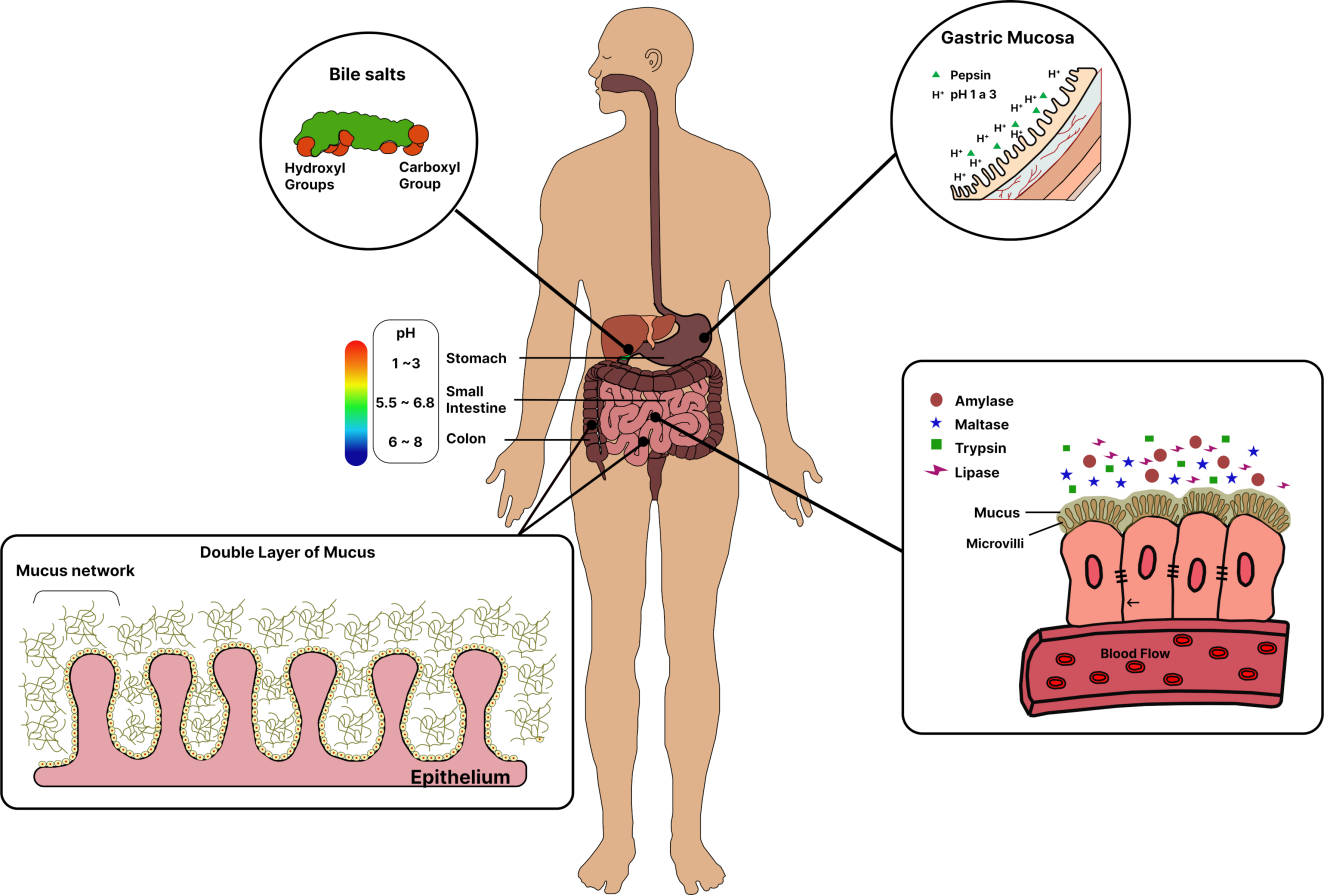
Physiological barriers along the gastrointestinal tract that pose challenges for oral drug delivery. Created with Figma.com (https://figma.com/).

Despite their advantages, drug candidates for oral administration face several limitations, as shown in Figure 1, including chemical instability caused by the gastric environment, low bioavailability, and poor permeability across barriers in the gastrointestinal tract (GIT), such as mucus and the intestinal epithelium [2–3]. The complex biochemical environment includes pH variations (ranging from 1 to 2.5 in the stomach to 7 to 8 in the colon), metabolizing enzymes (such as pepsin, lipase, peptidase, and amylases), and surfactants like bile salts and those produced by the liver. These factors can alter the structural integrity of drugs [4].

Orally administered drugs (mostly absorbed in the lumen of the GIT must pass through the mucus layer, located between the epithelial tissue and the lumen, to reach the epithelium, mucosa, and blood or lymphatic capillary walls. Since mucus is continuously secreted along the GIT, it is subsequently eliminated due to the cellular renewal process [5].

Mucus is a complex hydrogel comprising proteins, carbohydrates, lipids, salts, antibodies, bacteria, and cellular debris. Mucins are the primary protein component of mucus, secreted or cell-bound, which form a viscoelastic gel through entanglement and cross-linking. These properties contribute to the protective and lubricating functions of mucus. These functions are performed through steric, electrostatic, and hydrophobic interactions with various substances [6]. Mucin is an anionic glycoprotein that plays a pivotal role in determining the thickness and structure of the mucus layer. Consequently, its barrier function and permeability to drugs are also influenced. As a consequence, positively charged substances are attracted to mucin by electrostatic forces and retained within the mucus layer which acts as a barrier that must be overcome in the oral administration of drugs [7].

Another intrinsic physical barrier of the GIT is the intestinal epithelium (Figure 1), which consists of a single layer of epithelial cells, including enterocytes and secretory cells. Drug passage through the epithelium occurs via the transcellular and paracellular pathways, as well as through M cells located on the surface of Peyer’s patches [8]. Positioned under the mucus layer, the intestinal epithelium plays crucial roles in defending against hostile contents, selectively absorbing nutrients, and maintaining homeostasis within the GIT. The diverse cells of the intestinal epithelium can be explored as therapeutic targets for oral absorption, given the significant roles they play in the epithelial barrier [9].

Nanotechnology is a field that focuses on the study and production of nanometric particles. Materials reduced to this scale exhibit alterations in their physicochemical properties, enhancing their interactions with molecular and cellular processes. Consequently, nanotechnology has diverse pharmaceutical applications, as the nanoencapsulation of drugs improves solubility and biodistribution while preventing undesirable interactions and degradation before reaching target tissues and cells [10]. Moreover, by overcoming the biological and chemical barriers of the body, nanotechnology increases the efficiency and effectiveness of therapeutic and diagnostic regimens, offering less invasiveness and greater biocompatibility [11].

Polymeric nanoparticles (PNs) have been studied for their potential in the oral delivery of insoluble drugs and biological products [12]. Peptides, such as GLP-1 receptor agonists [13], nucleic acids such as RNA [14], insulin [15], and antigens [16] have been investigated. These nanoparticles (NPs) preserve the bioactivity of the active compound during gastric transit and facilitate permeation through mucus and absorption by epithelial cells, enabling targeted delivery. In essence, PNs offer superior performance in protecting, targeting, and enhancing both the therapeutic payload and intestinal permeability [17].

PNs represent a viable strategy for overcoming GIT barriers in oral drug delivery. The polymeric composition allows for diverse and sophisticated designs, with primary advantages including the ability to control size, shape, and surface charge. Furthermore, polymers enable the incorporation of numerous chemical and biological functionalities, rendering NPs able to target specific locations within the body. They can also be directed to individual cellular compartments and exhibit responsiveness to stimuli associated with particular physiological environments, thereby achieving highly efficient drug release [18]. This interaction with biological systems without inducing negative or toxic effects is attributed to their biocompatibility and biodegradability [19].

Surface design plays a pivotal role in PNs for oral use, as it dictates their behavior during absorption by interacting with food, digestive enzymes, bile salts, electrolytes, and mucus. Polymers enable diverse surface functionalities tailored to therapeutic demands, including adhesive, bioinert, or charge-conversion functionalities that modify zeta potential and hydrophilic properties, among others. Therefore, different polymer surfaces impact the NPs’ fate in the GIT in a different way [20].

Polymers are classified as either natural, derived from natural products, or synthetic, chemically synthesized from monomers. Examples of natural polymers include chitosan, alginate, and hyaluronic acid. Synthetic polymers are classified based on their chemical composition and include polyesters, polyamides, polyethylene glycol derivatives, and responsive polymers, among others. The most frequently used polymers as oral nanocarriers include poly(lactic-*co*-glycolic) acid (PLGA), polycaprolactone (PCL), and methacrylates. Strategies to enhance drug entrapment and improve interactions with biological systems often combine synthetic and natural polymers, with the latter serving as a coating to protect against degradation [21].

Considering the advantages of nanoparticulate systems in drug delivery, this review explores recent advances in the use of polymers for developing oral NPs, focusing on innovative research published in the past decade. It highlights the key characteristics of PNs and their preparation methods. The synthesis processes, chemical structures, and functions of major polymers are detailed, along with the mechanisms of active substance internalization and release within the body. Special attention is given to how PNs have been applied to enhance the oral delivery of peptides, nucleic acids, poorly soluble drugs, and small molecules. Additionally, current strategies for administration and bioavailability enhancement are discussed. The objective of this study is to provide a comprehensive theoretical foundation for employing polymers as nanocarriers in oral drug delivery.

### 2 Polymeric nanoparticles: a viable option for oral administration

The biocompatibility and biodegradability of polymers have made PNs a topic of growing interest in light of their small size and unique physicochemical properties [22–23]. These systems offer advantages for drug delivery, including the ability to protect labile compounds, control release kinetics, improve drug solubility and stability, enhance oral bioavailability, and enable site-specific targeting and combination therapies [24].

PNs are stable colloidal nanostructures formed from synthetic or natural polymers, characterized by synthetic versatility, which allows for customization based on the requirements of the final formulation. Biopolymers can undergo chemical derivatization to obtain specific properties, while synthetic polymers are synthesized from their corresponding monomers, resulting in diverse structures and applications [25]. PNs are viable options for smart drug delivery using responsive and stimulating polymers, which release molecules in response to internal signals (e.g., oxidation or reduction, enzymatic activity, or low acidity) or external stimuli such as light and temperature [26–27]. This delivery of active ingredients relies on differential biochemical changes, facilitating the development of more precise therapies with improved control over site specificity and drug release [28].

**Figure 2:**
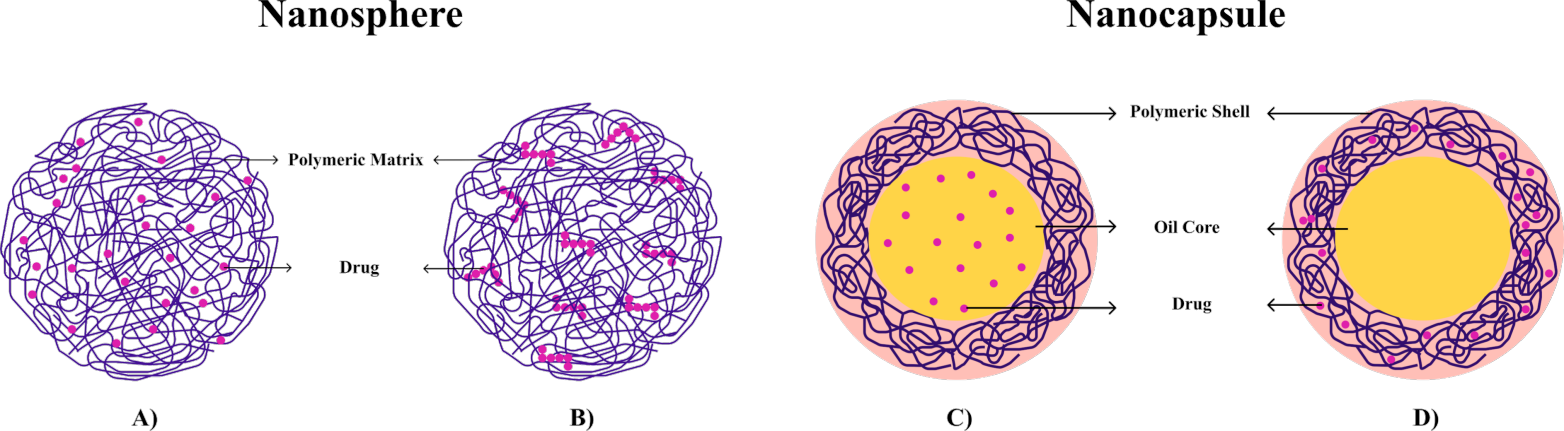
Representation of nanospheres and nanocapsules. (A) Nanosphere with the drug retained within the polymeric matrix; (B) nanosphere with the drug adsorbed or dispersed in the polymeric matrix; (C) nanocapsule with the drug dissolved in the oily core; and (D) nanocapsule with the drug adsorbed onto the polymeric matrix. Created with Figma.com (https://figma.com/).

The absorption of PNs depends on their size, surface characteristics, and morphology, which may exhibit distinct features, classifying them as either nanocapsules or nanospheres. Nanocapsules consist of an oily core where the drug is encapsulated (Figure 2C), surrounded by a polymeric shell that regulates its release profile; alternatively, the drug may be adsorbed onto the polymeric wall (Figure 2D). In contrast, nanospheres are formed when the drug is either adsorbed onto the surface of the polymeric matrix (Figure 2B) or retained inside it (Figure 2A), resulting in a uniform dispersion of active compounds within the polymer [29–30].

The encapsulation of drugs in nanocapsules and nanospheres correlates with their biopharmaceutical characteristics, which are directly linked to oral absorption. Amidon et al. [31], through studies correlating solubility and permeability in the cell membrane both in vitro and in vivo, demonstrated the importance of these characteristics for the pharmacokinetic performance of drugs and developed the “Biopharmaceutical Classification System” (BCS), which remains widely used today by regulatory agencies, research groups, and the pharmaceutical industry. The BCS classifies drugs into four categories, namely, class I (high solubility and permeability), class II (low solubility and high permeability), class III (high solubility and low permeability), and class IV (low solubility and permeability).

In this context, nanocapsules are particularly effective in encapsulating hydrophobic and lipophilic drugs due to the affinity of these compounds for the oily core. Drug release occurs through controlled diffusion across the polymeric layer and interaction with the core material. Conversely, nanospheres are capable of encapsulating either hydrophilic or hydrophobic drugs, depending on the physicochemical compatibility between the drug and the polymeric matrix. The distribution of the drug within the matrix is influenced by drug–polymer interactions, as well as by the type of polymer and the manufacturing method employed. Release from nanospheres is generally governed by a combination of polymer degradation and drug diffusion through the matrix. Additionally, when hydrophilic polymers are employed, swelling-induced release becomes relevant, wherein polymer chain relaxation significantly influences the diffusion rate and overall release kinetics [24,32–33]. From a pharmacokinetic perspective, the size, surface charge, and morphology of PNs can modulate their transit through the GIT, and such properties can be tuned during nanoparticle preparation. The choice of manufacturing method considers not only these parameters but also the physicochemical characteristics of the drug, the intended route of administration, the therapeutic target, and the polymer selected as the nanocarrier, which plays a critical role in determining the most appropriate fabrication technique. While drug classification by the BCS remains a reference in formulation development, the design of PNs expands the delivery possibilities for drugs in all four BCS classes by overcoming limitations related to poor solubility or permeability [34].

Among the various techniques used for preparing NPs, the polymerization of monomers or dispersion of preformed polymers are the most commonly used techniques. Methods based on the polymerization of monomers include emulsion polymerization, miniemulsion, microemulsion, and interfacial polymerization, which differ in initiation mechanisms and stabilization strategies. Emulsion polymerization, for instance, can occur in organic or aqueous continuous phases, with the latter offering a reduction of toxic solvent residues. Miniemulsion and microemulsion methods provide better control over particle size and distribution, although microemulsion polymerization often requires higher concentrations of surfactants and results in lower polymer chain yields. Interfacial polymerization, in contrast, is characterized by the reaction of two monomers dissolved in immiscible phases, leading to polymer formation at the interface [35–36]. Table 1 summarizes the characteristics of these methods and the most commonly used polymers.

**Table 1:** Description of polymerization-based methods for nanoparticle synthesis.

Polymerization method	Description	Polymers	Reference

Emulsion	- Rapid and scalable technique using organic or aqueous continuous phases - In the organic route, monomers are dispersed in non-soluble media and require surfactants to prevent aggregation - The aqueous route involves direct dissolution and initiation by high-energy radiation or free radicals - Polymerization kinetics depend on solubility and particle nucleation capacity	poly(methyl methacrylate) (PMMA); poly(alkyl cyanoacrylate)	[35–37]
Miniemulsion	Envolves the addition of a low molecular weight co-stabilizer and a high-shear device, distinguishing it from conventional emulsion polymerization while retaining the use of initiator, surfactant, monomers, and water.	poly(*n*-butyl cyanoacrylate), copolymers of acrylates	[38]
Microemulsion	- Produces fewer polymer chains due to selective droplet initiation - Requires high surfactant concentration and water-soluble initiator - Polymerization kinetics and component concentrations critically influence nanoparticle characteristics	PMMA, poly(styrene)	[2–4]
Interfacial	- Reaction between two monomers in immiscible phases at the interface	polyamide, polyurethane	[34]

Although polymerization-based techniques allow for precise tailoring of nanoparticle architecture, they present significant limitations that restrict their broader application, particularly in oral drug delivery systems, where biocompatibility and safety are critical. These methods frequently involve non-biodegradable monomers, generate non-biocompatible byproducts, and require extensive purification to eliminate potentially toxic residues, thereby complicating scalability and regulatory compliance. Moreover, the use of free radicals or ultraviolet light to initiate polymerization raises additional concerns regarding the stability and safety of the final product. In light of these challenges, the use of preformed polymers has gained prominence in nanoparticle synthesis, offering a more straightforward and biocompatible alternative particularly suited for pharmaceutical applications requiring materials approved by regulatory agencies such as the Food and Drug Administration (FDA) [29–30,39–40].

Given this context, PNs produced using preformed polymers have emerged as a technically and therapeutically advantageous option. Table 2 provides an updated overview of the most commonly used methods for the preparation of PNs employing preformed polymers. These methods typically begin with the formulation of an emulsifying system, a step common to all techniques, followed by the formation of NPs through a process that varies depending on the method, such as solvent precipitation or evaporation, which ultimately defines the nomenclature of each. The selection of suitable polymers is governed by their solubility profiles, interactions with solvents and drugs, and surface charge, which requires careful consideration to meet the therapeutic demands of oral delivery systems [41–42].

**Table 2:** Nanoparticle production methods by preformed polymers.

Method	Description	Advantages	Limitations	Type of PNs	Ref.

Nano- precipitation	- Created by Fessi and collaborators in 1989 - Interfacial deposition in which the transport of a solvent to a non-solvent promotes the dissolution of the polymer, leading to the growth of nuclei, crystals and nonoprecipitation - Organic phase formed by polymer, organic solvent miscible in water - Organic phase added drop by drop in the aqueous phase, with instantaneous formation of nanoparticle. Evaporation of the solvent - Due to its preparation method, it is also called desolvation and solvent displacement - When there is a miscible solvent and an immiscible antisolvent, it is called antisolvent precipitation	Low energy consumption; cost-effective, simple, reproducible, and scalable; suitable for hydrophobic compounds; single-step nanoparticle synthesis; applicable to different types of NPs	- Agitation rate impacts particle size - Not suitable for water-soluble molecules	nanosphere, nanocapsule	[43–44]
Emulsification/solvent evaporation	- First method developed for PNs preparation - Can be performed using single or double emulsion techniques - The polymer is dissolved in a solvent, and the mixture is emulsified in the aqueous phase using high shear force - In the second step, the solvent evaporates, causing polymer precipitation and PNS formation	simple, versatile and scalable; single step emulsion for hydrophobic actives; double or multiple emulsion for hydrophilic actives	- Better performance of liposoluble molecules - Coalescence of NPs during evaporation - High energy in homogenization - Nanocapsule only by double emulsion	nanosphere, nanocapsule	[32,45–46]
Emulsification/solvent diffusion	- The polymer is dissolved in an organic solvent saturated with water, creating an organic phase that is emulsified in an aqueous solution, resulting in solvent diffusion and PNs production - To precipitate the polymer, the solvent is diluted with water to enhance diffusion - The solvent is removed by evaporation or filtration and must be water-miscible - The aqueous solution must contain a surfactant, and the dilution phase consists of water	- Simple, reproducible, and scalable - Does not require a homogenizer - Encapsulates hydrophobic and hydrophilic molecules	- Elimination of large volumes of water from the suspension	nanosphere	[36,47]
Ionic gelation	- Used in the manufacture of NPs based on biopolymers, especially chitosan, and for protein encapsulation - Relies on electrostatic interactions between ionic polymers and cross-linking agents (ionic cross-linking) - The entire production process occurs under constant agitation - It begins with the dispersion of the active ingredient in a surfactant hydrocolloid solution to precipitate the hydrocolloid - Finally, cross-linking agents are added for hardening or stabilization, completing the PNs production	- Simple and cost-effective - Does not require organic solvents	- Low reproducibility - Research on polymers other than chitosan is limited	nanosphere	[10,48]
Coacervation	- Classified as simple or complex - Simple coacervation:self-assembly induced by desolvation or dehydration - Complex coacervation:occurs through interactions between oppositely charged polymers; influenced by pH, temperature, ionic strength, charge density, and the molecular weight of polyelectrolytes	- Simple - Does not require organic solvents	- Difficulty in scaling - Particle aggregation	nanosphere, nanocapsule	[49–50]
Dialysis	- The polymer is dissolved in an organic solvent and kept in a dialysis tube or membrane, dialyzed against a non-solvent - The displacement of the solvent in the membrane (or tube) leads to polymer aggregation due to increased surface tension, forming NPs	- Simple and cost-effective	- Large volume of counter dialysis medium - Long process - Early release of NPs	nanosphere	[30,51]
Spray drying	- Used as a final step for transformation into dry powder or as a direct manufacturing method - Single-step and continuous process	- Simple and fast - Reproducible and scalable	- Aggregation or particle growth - Low encapsulation efficiency for hydrophilic drugs	nanosphere	[52–54]

These strategies involve dissolving or dispersing pre-existing polymers in appropriate solvents, followed by NP formation through approaches such as nanoprecipitation, solvent diffusion-emulsification, solvent evaporation, ionic gelation, and dialysis. Each technique is intrinsically linked to specific polymer classes, depending on their physicochemical properties and solubility behavior. Nanoprecipitation is frequently employed for synthetic polymers such as PLGA and PCL, which are soluble in organic solvents and undergo controlled precipitation in aqueous media, enabling efficient encapsulation of hydrophobic drugs [55].

Chitosan, a natural polymer, predominantly forms NPs via ionic gelation, where interactions with multivalent counterions create stable polymeric networks without the use of organic solvents, thereby ensuring high biocompatibility and minimal toxicity. Similarly, alginate NPs are often obtained through emulsification/solvent diffusion techniques, facilitated by its water solubility and gel-forming capacity in the presence of divalent cations that support the encapsulation of hydrophilic drugs. Dialysis methods, in turn, are particularly suited for amphiphilic copolymers and polyethylene glycol, as these materials tend to self-assemble into micelles or stable nanostructures when exposed to concentration gradients in aqueous solutions. Preformed polymer-based methods stand out for their simplicity, reproducibility, and regulatory compliance, reinforcing their suitability for safe and effective oral nanoparticle delivery [44,51,56].

### 3 Polymers and their absorption mechanisms in the body

#### 3.1 Physical properties of polymers and their relationship with biodegradation in the body

Analyzing the Greek etymology of the word “polymer” provides insight into its definition. The term “polymer” is derived from “poly”, meaning “many” and “meres”, meaning “parts” or “units”. “Monomer”, in turn, comes from “mono”, meaning “one”, referring to a single molecule. Polymers, therefore, are macromolecules formed by smaller units, the monomers, which are covalently bonded to each other and produced through polymerization reactions such as addition, condensation, ring-opening polymerization, emulsion, precipitation, and others [57]. Monomers determine the decomposition products of polymers in the body and, consequently, their toxicity and biocompatibility. This is why synthesis methods are developed to control the rapid and reversible activation and deactivation of propagation chains. As a result, polymers with low polydispersity and controllable molecular weights are obtained, generating unimodal NPs with increasingly functional designs and satisfactory release profiles [58–59].

The intrinsic characteristics of polymers influence biodegradation and drug release behavior. Parameters such as crystallinity, glass transition temperature (*T*_g_), solubility, and molecular weight affect the polymer matrix and the behavior of incorporated molecules. This is because each polymer has unique spatial attributes, with monomeric structures and functional groups, hydrophobic or hydrophilic, that determine its properties [60].

The nature of monomers correlates with polymer crystallinity, which, in turn, defines mechanical strength, swelling, hydrolysis, and biodegradation rates. Crystallinity is associated with ordered molecular structures that exhibit intense interactions and steric hindrance. In this context, it can be inferred that low-molecular-weight molecules form crystals more easily and, consequently, have a slower drug release profile. Conversely, in polymers with greater porosity, crystallinity has a reduced impact on molecule release [61].

The influence on compound diffusion can be attributed to the polymer’s molecular weight, which affects the formulation’s behavior, aggregation, and architecture. Degradation products form more rapidly in polymers with lower molecular weight, as these exhibit a high elastic modulus, creating a deformable matrix that expands pores due to osmotic pressure [57]. This, in turn, influences the elimination, phagocytosis, and biological activity of PNs intended for oral administration.

Another factor affecting the polymer’s physicochemical properties is its *T*_g_, which determines the point at which the polymer transitions from a rigid, glassy state to a more flexible, rubbery state as the mobility of the polymer chains increases with rising temperature. Above *T*_g_, the polymer becomes rubbery, leading to increased rates of water and drug mass transfer throughout the entire polymer matrix. However, in NPs, a balance between amorphous and crystalline states is necessary to optimize mechanical strength and drug release rates. The glass transition temperature reflects the polymer chain’s permeability and mobility, influencing its susceptibility to enzymatic degradation. Consequently, copolymers with hydrophobic and hydrophilic segments are widely used in NPs to ensure more predictable release rates [28,62–63].

The hydrophobicity and hydrophilicity of polymers are directly related to their in vivo stability, influencing the pharmacokinetics and biological distribution of NPs. Increased hydrophilicity determines the degradation rate and their recognition by the reticuloendothelial system, preventing adsorption and nonspecific protein interactions. Furthermore, it enhances steric repulsion, reducing opsonization and activation of the complement system. Consequently, coating the nanoparticle surface with hydrophilic polymers decreases hydrophobicity and enhances its stealth properties [64].

Polymeric coating strategies are instrumental in preventing interactions with mucin through hydrophobic, electrostatic, and hydrogen bonds, while also increasing mucus penetration by modulating cellular absorption and reducing particle entrapment [65–66]. Maisel et al. [67] demonstrated that hydrophobic mucoadhesive particles adhere to mucus, leading to aggregation and limited distribution along the GIT after oral administration. Conversely, non-mucoadhesive particles can penetrate the mucus of the small and large intestine, spreading across nearly the entire tissue surface and facilitating drug delivery for systemic applications. This behavior occurs because surface properties influence polymer–mucus interactions, altering the entanglement density of mucin chains and consequently modifying the aggregation degree of the mucin network through various interactions [68–69].

Surface coating techniques play a key role in the interaction of PNs with the biological environment. These strategies involve the physical adsorption or covalent attachment of hydrophilic polymers (e.g., polyethylene glycol (PEG) or polysaccharides) or mucoadhesive agents such as chitosan, which can enhance colloidal stability, promote mucus permeation, and protect the nanocarrier from enzymatic degradation in the GIT. Such coatings have been explored to improve the pharmacokinetic performance of PNs by modulating their interactions with mucus and the intestinal epithelium. The selection of coating material and technique is guided by the therapeutic goal, the physicochemical properties of the core polymer, and the nature of the encapsulated drug. Section 4 of this review provides a detailed discussion of examples of polymeric coatings on NPs intended for oral administration, using both synthetic and natural polymers [70–71].

Polymer properties such as *T*_g_, solubility, crystallinity, and molecular weight are interrelated and have a significant impact on the permeability of NPs. Consequently, these properties affect interactions with mucin, pH, ionic strength, and physiological variables such as mucus thickness, clearance, and nanoparticle size, which collectively shape the mucoadhesive profile of polymers, enhancing cellular absorption [72–73]. Thus, the physicochemical characteristics of polymeric nanocarriers confer protective effects that prevent enzymatic metabolism prior to absorption, forming a barrier that remains stable for a specific duration until the encapsulated agent is released [74].

#### 3.2 Absorption mechanisms of polymeric nanoparticles

Cellular absorption occurs after overcoming the adverse conditions of the GIT, as nanocarriers enter the bloodstream to exert their therapeutic effect. This process can occur through various mechanisms, including paracellular transport, the M cell-associated pathway, and endocytosis.

Paracellular transport involves NPs reaching the bloodstream through the spaces between intestinal epithelial cells. Although these junctions are tight, they provide a useful route for administering macromolecules like insulin. Chitosan, as a polymeric nanocarrier, has shown satisfactory results by increasing permeability through the reversible opening of these intercellular tight junctions [75] as described in Table 2 and discussed in Section 4.1.1.

M cells are characterized by invaginations in their membrane, which facilitate the selective entry of substances into the lymphatic tissue beneath the intestinal mucosa via active transport. This pathway offers an alternative to first-pass metabolism and helps reduce toxicity. Moreover, NPs using this route are intercepted by immune cells such as macrophages and dendritic cells, making them suitable for oral vaccine administration (see Table 2) by promoting mucosal immunity activation [16,76].

**Figure 3:**
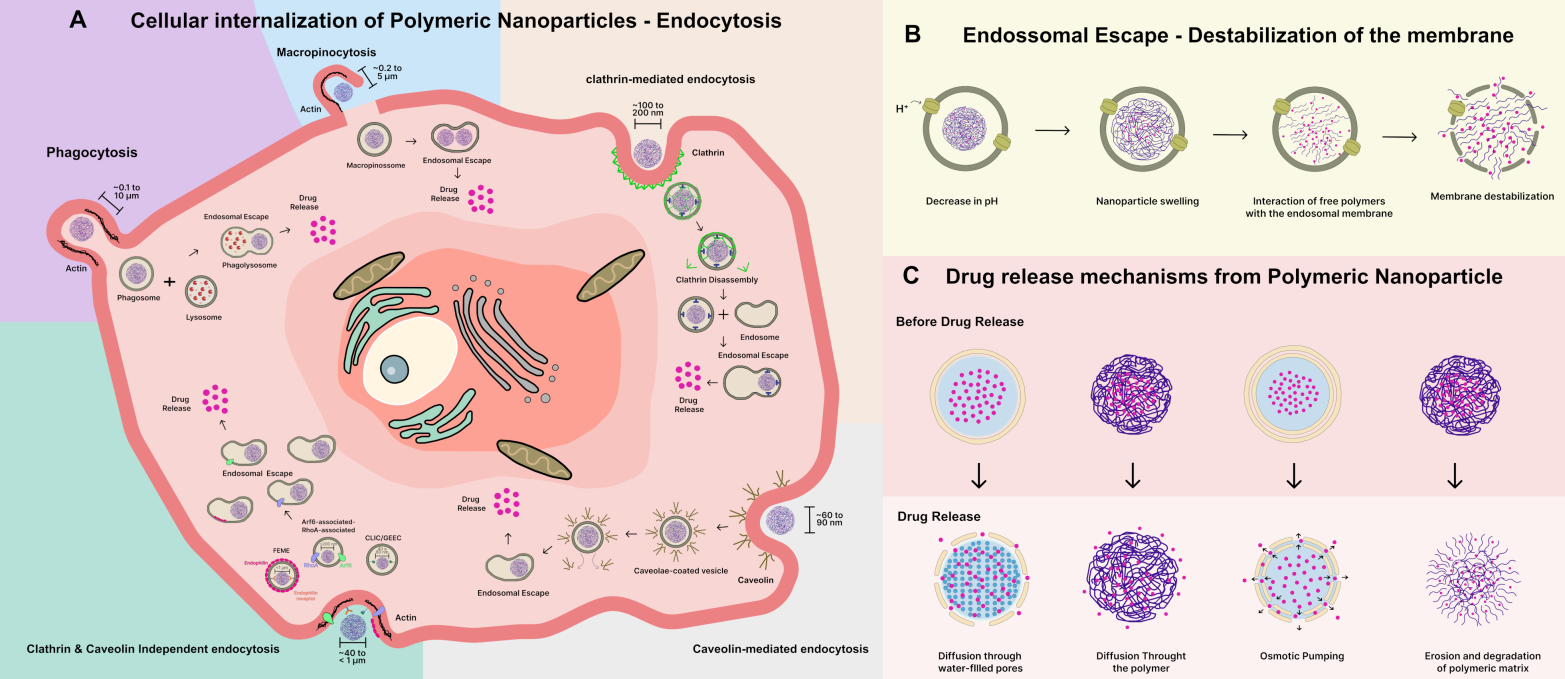
Representation of the cellular internalization pathways of polymeric nanoparticles (PNs), factors modulating their uptake, intracellular escape, and release of active ingredients. (A) Mechanisms of cellular uptake via endocytosis: phagocytosis, macropinocytosis, clathrin-mediated endocytosis (CME), caveolin-mediated endocytosis (CvME), and clathrin- and caveolin-independent endocytosis. (B) Endosomal escape through membrane destabilization (C) Mechanisms of drug release from polymeric nanocarriers. Created with Figma.com (https://figma.com/).

Endocytosis is the primary pathway through which NPs enter cells. Hydrophobic polymeric NPs often exhibit stronger interactions with the lipid bilayer, which may facilitate their internalization through endocytic mechanisms. In contrast, hydrophilic NPs tend to adsorb onto the cell membrane surface before being taken up via endocytosis [77]. Once internalized, NPs can be trafficked through various intracellular compartments (including endosomes and lysosomes) and in some cases, may escape into the cytosol, which is particularly relevant for therapeutic applications that require cytoplasmic or organelle-specific drug delivery. In this context, endocytosis is commonly categorized into two types, that is, phagocytosis and pinocytosis, as illustrated in Figure 3A. Phagocytosis involves the uptake of particles larger than 500 nm that bind to specific surface receptors (such as Toll-like, mannose/lectin, and scavenger receptors) on phagocytic cells. These particles are internalized through the plasma membrane, forming a cup-shaped invagination that develops into phagosomes. These phagosomes subsequently fuse with lysosomes, where the contents undergo acidic degradation [78–79].

Internalization of PNs by pinocytosis can occur through macropinocytosis, clathrin-mediated endocytosis (CME), caveolin-mediated endocytosis (CvME), and clathrin- and caveolin-independent endocytosis. Macropinocytosis is a type of endocytosis that depends on the solute concentration around the cell and is characterized by membrane protrusions induced by growth factors, bacteria, viruses, and necrotic cells. These protrusions either collapse into endocytic vesicles or retract into the membrane, fusing with it and forming macropinosomes [80].

CME involves interactions between clathrin-associated adaptors and sorting proteins, forming coated pits along the plasma membrane. These pits undergo disassembly via dynamin, leading to the formation of vesicles that are transported to endosomes for subsequent degradation. NPs smaller than 200 nm with a positive surface charge, as well as growth factors and transferrin, are internalized through CME, enabling targeted therapy [81].

The dimeric protein caveolin-1 is the primary agent in CvME, playing key roles in cell signaling, lipid regulation, and vesicular transport. It also defines the characteristic flask-like shape of vesicles and is located on the cytosolic side of the membrane. Similar to CME, dynamin is responsible for vesicle formation; however, in this pathway, vesicles fuse with caveosomes, avoiding lysosomal degradation. This mechanism is the main advantage of this internalization pathway, making it a suitable alternative when NPs are sensitive to degradation. Albumin, folic acid, peptides, and NPs smaller than 50 nm with surface charges between +15 and −15 mV are preferentially internalized via CvME. Some NPs follow clathrin- and caveolin-independent endocytosis pathways, which involve distinct uptake mechanisms, often dependent on cholesterol. These alternative pathways remain poorly understood [82]. Figure 3A illustrates all these mechanisms of cellular internalization of PNs.

After endocytosis, NPs become confined within intracellular compartments. However, as this is not the site of action for most encapsulated molecules, endosomal escape occurs (Figure 3B). This process involves the degradation pathway, where NPs follow the endosome–lysosome route post-endocytosis, with the acidity and lysosomal enzymes facilitating the release of the encapsulated constituents [83–84]. Endosomal escape is crucial for the release of nanoencapsulated actives, and PNs achieve this by destabilizing the membrane (Figure 3B). This process involves nanoparticle disassembly triggered by a pH decrease, leading to membrane disruption through interactions between the hydrophobic regions of the free polymer and the lipid membrane. Consequently, lipophilic polymeric moieties and adjustments in the disassembly pH can optimize this mechanism, which must occur during the endo/lysosomal pathway to minimize cytotoxic effects. This highlights the significance of polymeric chemical functionality as a key factor for escape efficiency [85].

Once endocytosis followed by endosomal escape occurs, drug release from the polymer matrix can proceed through various mechanisms after in vivo administration. In this context, water is absorbed by the polymer, leading to pore formation, which results in the creation of degradation products that dissolve in the release medium. Increased contact with water triggers hydrolysis, producing acids that catalyze degradation, thus enlarging the pores, a phenomenon known as swelling, which is particularly relevant in systems based on hydrophilic polymers. The mobility of the polymer chains, which is influenced by their *T*_g_ (decreasing as the polymer molecular weight increases), has the potential to close the pores and regulate the release of the active ingredients. This release can occur through diffusion via water-filled pores, diffusion through the polymer matrix, osmotic pumping, and erosion [10,86].

The release of the encapsulated agent refers to the transport of the molecule from its initial position within the polymer matrix to the external surface for subsequent release into the surrounding environment. In this context, release may occur through four mechanisms (Figure 3C), namely, drug diffusion through water-filled pores, diffusion through the polymer matrix, osmotic pumping, and erosion.

Release via diffusion through water-filled pores depends on a concentration gradient. Water absorbed by the NPs penetrates the polymer matrix, and progressive hydration leads to the formation and expansion of water-filled pores, the structure of which evolves with matrix degradation. This facilitates drug diffusion as the pores become larger and more numerous. Conversely, in diffusion through the polymer matrix, release occurs predominantly by molecular diffusion, where the release rate is governed exclusively by the permeability and thickness of the membrane, and remains constant and independent of concentration gradients [28].

In addition to diffusion, drug transport through water-filled pores may also occur by convection. The porosity of the nanostructure promotes water influx, which is induced by osmotic pressure, facilitating drug release via osmotic pumping. Diffusion-controlled release is directly influenced by the effective diffusion coefficient of the drug within the polymer matrix [10].

Erosion, as a release mechanism, involves the degradation of the polymer matrix, altering the nanostructure in two ways: surface and bulk erosion. In surface erosion, the polymer begins to degrade at the outer layer of the matrix and progressively advances inward, with the degradation rate exceeding that of water penetration. In bulk erosion, water rapidly penetrates the entire polymer structure, promoting homogeneous degradation of the matrix. This type of erosion exhibits less predictable behavior and reduces the protective capacity of the carrier [28,87].

Mathematical models are also employed to understand the drug release mechanism from the polymer matrix to the external surface and, consequently, to its site of action. This is achieved through kinetic analysis using models such as Korsmeyer–Peppas, Higuchi, zero-order, Lindner–Lippold, Ritger–Peppas, Peppas–Sahlin, Weibull, and the Monte Carlo technique, incorporating both Fickian and non-Fickian processes, among others. Each particle follows a release pattern that involves complex processes and varied interactions with the cellular environment. Additionally, the release is influenced by factors ranging from polymer properties to cellular internalization mechanisms [88].

The absorption of PNs involves complex and multifaceted mechanisms, including overcome GIT barriers and intracellular release processes. Each absorption route offers specific advantages and challenges that must be addressed based on the therapeutic application [16]. In this context, the chemical functionality of polymers, their interaction with cell membranes, and controlled release dynamics are critical for drug delivery. Understanding these mechanisms and developing optimized nanocarrier systems are essential to enhance therapeutic efficiency, minimize adverse effects, and expand the potential for oral administration of PNs.

### 4 Polymer based nanoparticles for oral administration

#### 4.1 Nanoparticles: oral administration based on natural polymers

Nanotechnological systems derived from natural polymers found in biological species, such as proteins and polysaccharides, are referred to as biopolymeric NPs. Examples of natural polymers utilized in these NPs include casein, zein, hyaluronic acid, alginate, and chitosan [89].

**Figure 4:**
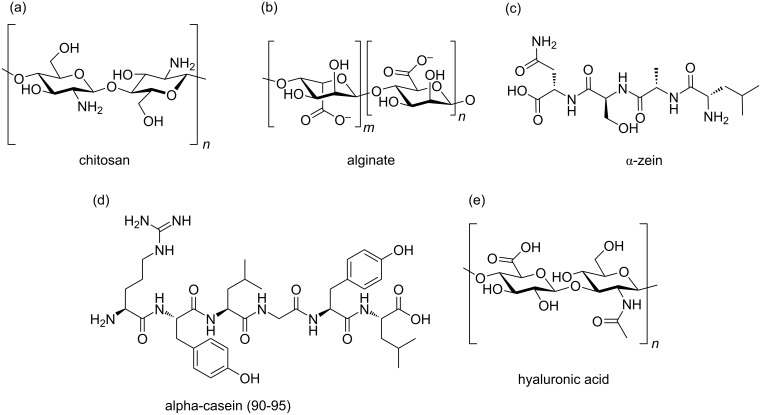
(a–e) Molecular structures of natural polymers widely used in nanoparticles for oral drug delivery. (c) Schematic representation of a segment of the α-zein fraction, the most abundant portion of zein, which constitutes the majority of this storage protein. (d) Schematic representation of α-casein, the main fraction of casein, illustrating its generic polypeptide nature. Created with Figma.com (https://figma.com/).

Biopolymers are macromolecules composed of repeating monomers linked by covalent bonds [90]. These materials are highly attractive as vehicles for oral drug delivery systems due to their strong mucosal adhesion properties and their ability to enhance paracellular transport of drug molecules. Additionally, they are easy to chemically modify, non-toxic, safe, biocompatible, and capable of regulating biodegradation in the body [91]. Figure 4 illustrates the polymers discussed in this section, highlighting those most commonly used for oral administration.

The abundance of functional groups in natural polymers enables modifications to enhance molecular target specificity, allowing for adaptation to specific therapeutic targets. Furthermore, their responsiveness to stimuli, particularly pH, makes them ideal for oral applications, given the diverse chemical environments of the GIT. Biocompatibility, low toxicity, and cost-effectiveness further support the use of biopolymers as nanocarriers for drug delivery.

**4.1.1 Chitosan.** Chitosan is the second most abundant natural polymer and one of the most extensively studied in biomedical sciences. Analysis of the studies presented in Table 3 confirms that it is among the most frequently utilized polymers in PNs for oral drug delivery. Structurally, chitosan (Figure 4a) is a linear polysaccharide composed of ᴅ-glucosamine monomers linked via β-(1→4) bonds [92–93].

**Table 3:** Studies using natural polymers for oral administration nanoparticles.

Polymer	Drug and BCS	Therapeutic class	Preparation technique	Ref.
Objective Results (Release, In vitro test, In vivo test)				

Hyaluronic acid	insulin (N/A^a^)	hypoglycemic agent	dialysis	[94]
Objective: Prepare mucoadhesive and pH-responsive NPs investigating the impact of the molecular weight of hyaluronic acid for oral insulin delivery. Release: Stability of PNs at acidic pH and insulin release were influenced by the molecular weight of hyaluronic acid. In vitro test: Improved antimucin adhesion, higher intestinal epithelium permeability, and deeper penetration in 2 h with lower molecular weight hyaluronic acid PNs. All PNs reduced insulin aggregation. Cell viability > 80%. No significant difference in PNs uptake. In vivo test: All PNs increased insulin bioavailability, with emphasis on those with hyaluronic acid with higher molecular weight, as it demonstrated better glycemic control.				

Hyaluronic acid	doxorubicin (III)	antineoplastic	dialysis	[95]
Objective: Develop PNs of hyaluronic acid copolymers and monoglycerides and diglycerides of fatty acids for oral delivery of doxorubicin, aiming to improve intestinal retention and increase lymphatic absorption. Release: Fatty acid monoglyceride PNs released doxorubicin more rapidly (55% in 12 h). In vitro test: PNs exhibit enhanced cytotoxicity in cancer cells. Cellular internalization occurs by clathrin- and caveolin-mediated endocytosis and macropinocytosis. Cell viability >85%. Fatty acid chain lengths in PNs contribute to their ability to cross Caco-2 monolayers In vivo test: Increased plasma concentration and area under the curve compared to free drug.				

Hyaluronic acid	deferoxamine (N/A^a^)	iron chelator	sonication	[96]
Objective: Synthesize and characterize hyaluronic acid PNs conjugated to deoxycholic acid (DOCA) and taurocholic acid (TCA) to improve oral delivery. Release: – In vitro test: Absence of cytotoxicity. Bile acids improved cellular uptake and PNs showed better permeation in the Caco-2 monolayer when compared to the free drug. Transcellular absorption via endocytosis mediated by hyaluronic acid. In vivo test: –				

Alginate; dextran	insulin (N/A^a^)	hypoglycemic agent	ionotropic gelation	[97]
Objective: Improving intestinal retention through PNs functionalized with immunoglobulin G. Release: No release occurred at pH 1.2. 70% in 2 h at intestinal pH. In vitro test: Absence of cytotoxicity. Functionalized PNs permeated CaCo-2 cells more rapidly and extensively. In vivo test: –				

Alginate; chitosan	lactoferrin (N/A^a^)	antibacterial immunomodulatory	nanoprecipitation ionic gelation	[98]
Objective: Evaluate the potential therapeutic activity that lactoferrin-loaded PNs may have in inflammatory osteoarthritis. Release: Controlled release of the protein over a 24 h time frame. In vitro test: Absence of toxicity. Increased nuclear activity by 156.87 ± 13.7%. Reversed mitochondrial depolarization with formation of aggregates. Demonstrated apoptotic activity. In vivo test: PNs demonstrated antiarthritic and disease-modifying activity.				

Alginate; chitosan	curcumin (IV)	Anti-inflammatory/ antioxidant	polyelectrolytic complexation	[99]
Objective: Develop and evaluate chitosan and alginate PNs with curcumin, dispersed in polymeric films for use in antimicrobial photodynamic therapy with oral biofilms. Release: Curcumin release was limited by diffusion in the polymeric matrix (*k* = 6.15). Films exhibited faster release (*k* = 29.45). In vitro test: The association of PNs with alginate oral films preserved mucoadhesion and reduced by 3 log10 CFU/mL against *Streptococcus mutans*. In vivo test: –				

Casein	curcumin (IV)	Anti-inflammatory/ antioxidant	coacervation	[100]
Objective: Investigate low-cost encapsulation, without organic solvents, exploiting the pH-dependent solubility of curcumin and the self-assembly of sodium casinate (NaCas). Release: First-order release with nanoparticles stable for 48 h at pH 7.0. Higher curcumin content accelerated release by faster replacement of casein with Tween^®^ 20. In vitro test: Enhanced antiproliferation activity against HCT-116 and BxPC3 cells when compared to free curcumin. In vivo test: –				

Casein	quercetin (IV)	Anti-inflammatory/antioxidant	coacervation	[101]
Objective: Optimize the preparation of casein NPs loaded with quercetin and evaluate their efficacy in oral absorption and bioavailability in Wistar rats. Release: pH-independent release: PNs without β-cyclodextrin released 80% of quercetin in 4 h. PNs with β-cyclodextrin released 60% in 24 h. In vitro test: – In vivo test: The nanoparticle showed a 111-fold increase in the area under the curve compared to quercetin suspension.				

Casein	doxorubicin (III)	antineoplastic	ionic gelation	[102]
Objective: Producing casein PNs with doxorubicin by an economical and simple method, using calcium ions for their formation. Release: More efficient release at acidic pH (90% in 6 h) than at intestinal pH (60% in 6 h). In vitro test: Higher cellular uptake of the NPs and greater cytotoxicity due to better internalization. Casein PNs without drug do not induce cytotoxicity. In vivo test: –				

Casein	mequindox (IV)	antimicrobial	electrostatic complexation	[103]
Objective: Develop and characterize mequindox-containing casein PNs to improve their oral bioavailability. Release: PNs with slower and sustained release compared to the free drug; 90% release in 24 h regardless of the pH. In vitro test: – In vivo test: Casein PNs showed 4-fold higher *C*_max_ and 1.20-fold increased area under the concentration curve when compared to the free drug.				

Casein	enrofloxacin (II)	antimicrobial	coacervation	[104]
Objective: Optimize the formulation of casein PNs with magnetic and ultrasonic agitation, without organic solvents. Release: Two-step release in the first two hours explosively and then continuously for 24 h. Lower release at acidic pH. Pure drug released 92.46% in 2 h and 100% in 4 h at acidic pH. In vitro test: – In vivo test: PNs with *C*_max_ 2.6 times higher than the enrofloxacin suspension, as well as an area under the curve 3.8 times higher. *T*_max_ of 1 h for both. Increased average residence time of PNs.				

Lysine	insulin (N/A^a^)	hypoglycemic agent	electrostatic complexation	[105]
Objective: Develop a lysine platform for oral administration, aiming for loading, protection and efficient transport of proteins. Release: Hyaluronic acid coating significantly delayed insulin release at acidic pH compared to uncoated particle. In vitro test: Absence of significant cytotoxicity and high cell viability of Caco-2 cells Internalization by the classical uptake pathway with energy consumption. In vivo test: PNs coated with hyaluronic acid exhibited more intense and long-lasting fluorescence in the abdomen, indicating greater intestinal retention and absorption compared to free insulin.				

Chitosan	paclitaxel (IV)	antineoplastic	emulsification	[106]
Objective: Synthesize ceramide-chitosan (CS-CE) conjugate for stable PNs that efficiently transport hydrophobic molecules, such as Paclitaxel (PTX), orally. Release: After 48 h, 30% of PTX was released from CS-CE PNs, indicating sustained and efficient drug release. In vitro test: Time-dependent proportional uptake. In B16F10 cells, the PNs showed cellular uptake 7 times greater than free paclitaxel, as well as a superior result in the MCF7 cell line. Absence of cytotoxicity. In vivo test: Nanoparticle reached almost double the plasma concentration (*C*_max_ 47.54 ng/mL) in 0.75 h compared to PTX in Cremophor/DMSO, improving drug transfer from the gastrointestinal tract.				

Chitosan	epigallocatechin-3-gallate (N/A^a^)	antioxidant	ionic gelation	[107]
Objective: Develop genipin-cross-linked casein-phosphopeptide (CPP)-chitosan (CS) PNs (<300 nm) and evaluate their stability and release in the gastrointestinal tract. Release: Tuning the degree of genipin cross-linking in CPP-CS PNs allows controlled release of payloads at target sites. In vitro test: Stable PNs after incubation in simulated gastric media with the presence of enzymes. Inhibition of cell growth and maintenance of IC 50 values = 0.123 mmol/L in BGC823 cancer cells. In vivo test: –				

Chitosan	peptideo YY (N/A^a^)	therapeutic agent	polyelectrolytic complexation	[108]
Objective: Increase the concentration of the polyelectrolyte by complexation in miniemulsions, compare two cross-linking methods and investigate the pH-dependent release of the peptide after encapsulation. Release: More peptides were released at pH 4 and 7 due to swelling of the polyelectrolyte matrix, while it shrinks near neutral charge, limiting release. In vitro test: – In vivo test: –				

Chitosan	enoxparin (N/A^a^)	anticoagulant	ionic cross-linking	[109]
Objective: Investigating pH-sensitive and mucoadhesive PNs to improve the oral bioavailability of low molecular weight heparin (LMWH). Release: PNs with sodium tripolyphosphate showed faster release than those with hydroxypropyl methylcellulose phthalate in simulated gastric fluid but not in intestinal fluid. In vitro test: Acid stability of PNs. Thiol ligand increased permeation 1.86-fold in freshly excised carp intestine. In vivo test: Improvement of mucoadhesion in the intestinal mucosa of rats. Activated partial thromboplastin time was prolonged and oral bioavailability of heparin increased.				

Chitosan	docetaxel (IV)	antineoplastic	ionic gelation	[110]
Objective: Develop docetaxel (DTX) nanocapsules to target cancer cells and improve gastric permeation. Release: Nanocapsules, unlike pure DTX suspension, exhibited >80% sustained release for 24 h due to folate swelling. In vitro test: Concentration-dependent cytotoxicity; 80% cell viability. IC_50_ of 0.0427 µg/mL showing greater anticancer potential at lower concentrations. In vivo test: The area under the curve of the nanocapsule was 8.91 times greater than that of the free drug.				

Chitosan	exenatide (II)	hypoglycemic agent	ionotropic gelation	[111]
Objective: Using chitosan polymers (CS) to encapsulate exenatide, employing sodium tripolyphosphate (TPP) as a cross-linking agent, and coating the exterior with sodium alginate (ALG) to provide stability in acidic environments. Release: 90% release in 1 h in simulated intestinal fluid. In simulated gastric fluid it was less than 10% in 3 h. In vitro test: Absence of cytotoxicity in Caco-2, HT-29 and Raji B cells. Cell viability close to 100%. Increased adhesion and slowing down of the mobile phase of cell membranes. In vivo test: Exenatide reached peak faster subcutaneously (0.5 h) than orally (2 h), but oral bioavailability was only 9.16%.				

Chitosan	Insulin (N/A^a^)	hypoglycemic agent	ionic gelation	[15]
Objective: Developing hyaluronic acid-coated chitosan PNs to protect insulin in the gastric environment. Release: Coated nanoparticle reduced initial insulin release at pH 1.2 and controlled release rate, with 43% total release at pH 6.8. In vitro test: Cell viability 99%. Absence of cytotoxicity to Caco-2 cells. Cellular internalization of PNs by endocytosis. In vivo test: Subcutaneous insulin caused short-term hypoglycemia, whereas PNs provided prolonged glucose control, lasting longer at higher doses.				

Chitosan; alginate	lactoferrin (N/A^a^)	antimicrobial immunomodulatory	ionic gelation nanoprecipitation	[112]
Objective: Evaluate the antitumor efficacy of chitosan-calcium phosphate (ACSC) nanocarriers loaded with iron-saturated lactoferrin (Fe-bLf), coated with alginate, in in vitro tests and in a breast cancer xenograft model. Release: Initial burst at the end of 12 h. Sustained release up to 96 h at alkaline pH. 55% at acidic pH at the end of 96 h. In vitro test: Increased cellular uptake to 85% ± 1.6% in 6 h. Multicellular MDA-MB-231 tumor spheroids showed complete dissociation in 72 h and 96 h when treated with the unencapsulated Fe-bLf nanoparticle; the spheroid did not dissociate in the same period. Significant reduction in cell proliferation. In vivo test: Absence of tumor growth in mice fed with Fe-bLf PNs.				

Chitosan	letrozol (I)	antineoplastic	Emulsification, ionic gelation	[113]
Objective: Develop and characterize a novel self-assembled nanocarrier using a cross-linker under acidic conditions to enhance oral absorption. Release: Nanoparticle protects the hydrophobic drug from degradation by gastric juice and allows its release at the alkaline pH of the gastrointestinal tract. In vitro test: Absence of cytotoxicity in MC7-7 and PC-12 cells. MTT assay showed that the nanocarriers reduced cell growth by up to 37.3% ± 0.06% and overcame the low water solubility of letrozole by effectively penetrating cell membranes. In vivo test: –				

Chitosan	inteferon alfa (N/A^a^)	immunomodulatory	ionotropic gelation	[114]
Objective: Develop oral nanoparticle with interferon gamma and alpha. Release: – In vitro test: 100% cell viability. Moderate cell permeability. Tests with Caco-2: HT29-MTX cells indicated that the presence of mucin does not affect the permeability of PNs. PNs are not endocytosed by enterocytes. In vivo test: There was no significant difference in the concentration of IFNα in the plasma after subcutaneous application of this free form and oral administration of the same in nanoparticle form.				

Chitosan	rifampin (II)	tuberculostatic	ionotropic gelation	[115]
Objective: Investigating the efficacy of chitosan in a pH-responsive environment as a potential drug delivery vehicle. Release: 40% in 24 h of dialysis at acidic pH and 70% after 24 h. In vitro test: – In vivo test: –				

Chitosan; casein; dextran	astaxanthin (II)	antioxidant	ionic gelation	[116]
Objective: Develop ternary complex PNs to improve the aqueous dispersibility of astaxanthin and optimize its antifibrotic therapeutic effect. Release: – In vitro test: PNs with low toxicity to LX-2 cells. Viability greater than 90%. Improved antioxidant activity. Antifibrogenic effects in PNs loaded with 3% and 6% of the drug. In vivo test: –				

Trimethyl- chitosan; cysteine	shRNA; siRNA (N/A^a^)	antineoplastic	electrostatic complexation	[117]
Objective: Develop galactose-modified trimethylchitosan-cysteine (GTC) conjugates for oral delivery of therapeutic genes. Release: – In vitro test: Paracellular transport. Increased permeation of nanoparticle-loaded pDNA and siRNA promoting transcytosis in normal enterocytes and intestinal M cells. GTC 2 PNs showed 1.9 times the amount of pDNA and siRNA uptake in BEL-7402 human hepatoma cells compared to the other formulations. In vivo test: Orally administered PNs showed greater plasma and tumor distribution than free genes. Galactose-modified PNs showed greater tumor accumulation due to efficient internalization.				

Trimethyl- chitosan; hydroxypropyl methylcellulose phthalate	hepatitis antigen (N/A^a^)	vaccine	ionic gelation	[118]
Objective: Prepare trimethyl chitosan (TMC)/Hydroxypropyl methylcellulose phthalate (HPMCP) PNs by ionic gelation, cross-linking pH-sensitive HPMCP with TMC, evaluating its application in the oral administration of hepatitis B surface antigen (HBsAg). Release: HBsAg released from TMC/HPMCP PNs after 6 h of incubation at pH 2 did not exceed 25% of the protein, demonstrating physical stability in gastric media. In vitro test: – In vivo test: –				

Trimethyl- chitosan- cysteine	RNA (N/A^a^)	anti-inflammatory	ionic gelation	[14]
Objective: Optimizing siRNA nanocarriers to improve release kinetics, aiming to increase RNAi efficacy in several inflammatory diseases. Release: Two formulations showed rapid siRNA release and greater than 70% at pH 7.4. Other formulations showed a varied release profile and below 60% in 8 h. In vitro test: Restricted siRNA unwrapping. Absence of cytotoxicity in RAW 264.7 cells. PNs with cross-linker released siRNA more rapidly, achieving 75% gene silencing, but lower in macrophages. Transfection depends on the optimal timing of siRNA release In vivo test: Formulation 5 demonstrated greater efficacy in silencing genes and reducing acute inflammation in animal models, rapidly releasing siRNA in hepatic macrophages and inhibiting TNF-α production				

Zein	resveratrol (II)	antioxidant	nanoprecipitation	[119]
Objective: Develop PNs loaded with resveratrol to enhance its stability and oral bioavailability. Release: Resveratrol release from zein PNs exhibited an initial rapid phase followed by a plateau, with the total amount of drug released being higher in lyophilized formulations containing 10% sugar. In vitro test: Apparent toxicity of resveratrol decreased, with an increase in CC50 from 200 μM to values exceeding 1000 μM. Viability of both cell lines was close to zero. PNs protect the drug from metabolic events. In vivo test: –				

Zein	insulin (N/A^a^)	hypoglycemic agent	desolvation	[120]
Objective: Evaluate the efficacy of oral nanocarriers prepared from zein PNs coated with a poly(anhydride)-thiamine (GT) conjugate for insulin delivery. Release: Coating the PNs with the GT conjugate reduced insulin release, particularly in acidic pH, indicating more efficient control of the release profile. In vitro test: – In vivo test: PNs significantly reduced fat content in nematodes and provided prolonged drug release with a pharmacokinetic profile similar to subcutaneous insulin.				

Zein	insulin (N/A^a^)	hypoglycemic agent	desolvation	[121]
Objective: Evaluate the ability of two different zein-based NPs, with mucoadhesive or mucus-permeating properties, for oral protein delivery. Release: Insulin released almost completely in 22 h, regardless of pH, with the polyethylene glycol coating promoting a slightly slower release. In vitro test: Absence of cytotoxicity in Caco-2 cells and HT29-MTX cells. In vivo test: Nanoencapsulated with later peak concentration and smaller area under the curve compared to subcutaneous insulin, with the propylene glycol coating slightly prolonging this effect.				

Zein	docetaxel (IV)	antineoplastic	solvent evaporation	[122]
Objective: Develop and characterize glucose-modified zein PNs loaded with docetaxel (DTX) to enhance oral bioavailability and antitumor efficacy. Release: Sustained release profile in both PNs systems. In vitro test: Modification of glucose increased oral absorption and bioavailability of the nanoparticle by 2.19 times, with a mean residence time 8.07 h longer. In vivo test: Modification of glucose increased oral absorption and bioavailability of the nanoparticle by 2.19 times, with a mean residence time 8.07 h longer.				

Zein	mometasone (II)	corticosteroid	nanoprecipitation	[123]
Objective: Produce and characterize mometasone-loaded zein PNs for an oral strategy that increases the local concentration of the drug in the intestine. Release: The mometasone release from zein PNs was pH-dependent, with greater release in the intestinal than in the gastric buffer. In vitro test: PNs exhibit the same toxicity profile as the free drug. Cell viability > 70%, 63% cell permeability, and short and intermediate safety in CaCo-2 and HT29-MTX intestinal cells. Transport of PNs across the cell monolayer. In vivo test: –				

Zein	quercetin (IV)	anti-inflammatory antioxidant	anti-solvent precipitation	[124]
Objective: Investigate the efficacy of zein nanospheres and nanocapsules on the oral bioavailability of quercetin and its antihyperlipidemic effect. Release: Similar release (≈70%) at acidic and basic pH, suggesting that pH-independent release and nanoparticle type did not influence. In vitro test: Similar hydrophobicity values for nanocapsules and nanospheres with a higher effective diffusion coefficient for nanospheres as well as diffusivity in intestinal mucus. In vivo test: Nanospheres showed higher bioavailability (57%) than nanocapsules (25%).				

Zein	curcumin (IV)	anti-inflammatory antioxidant	anti-solvent precipitation	[125]
Objective: Develop and characterize carboxymethylpachymaran/zein PNs with curcumin by anti-solvent precipitation. Release: Curcumin release from coated PNs was slower (9.5%) due to protection against pepsin degradation. In vitro test: Good biocompatibility and some inhibitory activity on tumor cells. In vivo test: –				

Zein; chitosan; sodium caseinate	naringenin (II)	antioxidant	anti-solvent precipitation	[126]
Objective: Develop naringenin PNs in a matrix of zein, sodium caseinte and galactosylated chitosan for specific targeting to hepatocytes. Release: Similar gastrointestinal release profiles of PNs. In vitro test: Aggregation inside and outside the cell membrane significantly inhibiting triglyceride levels (*p* < 0.05). In vivo test: Naringenin-zein-sodium caseinate-chitosan and naringenin-zein-sodium caseinate-galactosylated chitosan PNs improved non-HDL cholesterol and HDL-C levels, with greater lipid reduction.				

Zein-casein, zein-lacto- ferrin, zein-PEG	N/A^a^	–	phase separation	[127]
Objective: Investigate surface composition effects on zein nanocarrier performance for oral delivery. Release: PEG has a faster release (higher hydrophobicity) than casein. In vitro test: Non-toxic nanocarriers internalized via energy-dependent macropinocytosis, with uptake reduced by 50% at lower temperatures, targeting lactoferrin receptors. In vivo test: Nanocarriers prolonged dye retention in the gastrointestinal tract compared to free dye. Zein-casein PNs showed greater retention.				

^a^N/A: Refers to substances to which the biopharmaceutical classification cannot be applied because they are biological or similar molecules.

The widespread use of chitosan can be attributed to the functional groups within its molecular structure, which confer a polycationic character. These groups are reactive and can be chemically modified through activation or cross-linking. The reactivity of the hydroxy and primary amine groups imparts complexing and chelating properties, enabling the production of chitosan derivatives with enhanced characteristics compared to the precursor polymer. For instance, trimethylation of the primary amino group increases its cationic nature, enhancing mucoadhesive properties. Thiolation of chitosan forms disulfide bonds with mucus glycoproteins and intra-chain, enhancing mucoadhesive properties. Other modifications include carboxylation, the addition of chelating agents, PEGylation, and lactose conjugation, which target specific functionalities to improve performance as a drug carrier [128–130].

Chitosan nanoparticles have been modified to overcome limitations associated with the delivery of hydrophobic and unstable drugs, such as rifampin. In the study by Motiei et al. [92], chitosan was functionalized with hydrophobic amino acids to form an amphiphilic inner core. This core was stabilized with a polyanionic cross-linker and coated with a pH-resistant shell, resulting in a multilayered structure. This system exemplifies how targeted chemical modifications can enhance the encapsulation capacity and physicochemical protection of sensitive drugs. Such strategies are particularly relevant for the development of PNs for oral administration, where resistance to hydrolysis and oxidation in the gastrointestinal tract is critical [131].

In this scenario, Ghosh et al. [115] encapsulated rifampicin in a chitosan nanocarrier and investigated its efficacy in a pH-responsive environment, ensuring rapid and more efficient (75%) delivery of rifampicin at intestinal pH, compared to the stomach environment. This behavior depends on the charge properties and the interaction between chitosan and sodium tripolyphosphate (TPP) molecules. At low pH, the amino groups become protonated, causing the molecule to adopt an extended conformation due to charge repulsion. The TPP molecule is also protonated, generating a lower charge density. At pH 4, both TPP and chitosan undergo deprotonation, with the protonation of chitosan being less affected, leading to a strong TPP–chitosan interaction that prevents rifampicin release. Conversely, at basic pH, the increased TPP charge neutralizes the charge density in chitosan, causing the collapse of the nanoparticle structure and the release of rifampicin [115].

The encapsulation of biological agents by NPs has become a reality and remains a field of constant interest, with advanced pharmacokinetic studies due to the use against diseases of global importance. The oral administration of these compounds presents a challenge due to the unstable environment in the GIT, which is subject to various types of degradation. Biopolymers are being studied to facilitate the administration of these macromolecules [132]. Interferon alpha (IFNα) is one such cytokine of therapeutic interest due to its immunomodulatory activity, which plays a key role in antitumor therapy by inducing cell apoptosis [133].

Building on this, Imperiale et al. [114] evaluated the pharmacokinetic profile of chitosan NPs loaded with IFNα and analyzed the cellular compatibility of the NPs in intestinal epithelial cells (Caco-2) and the human amnion-derived cell line (WISH). They also assessed the permeability of the NPs in mucin-secreting cells and determined the complete pharmacokinetic curve. The nanocarriers exhibited moderate permeability through the paracellular pathway, suggesting the ability to open epithelial tight junctions. The pharmacokinetic profile revealed IFNα bioavailability in plasma similar to that observed with subcutaneous administration. This study was a continuation of the research by Cánepa et al. [134], which demonstrated the stability of these formulations in the gastrointestinal tract, showing that chitosan as a carrier does not interfere with the biological activity of the protein, as evidenced by oral absorption in mice.

The versatility of chitosan was utilized by Yang et al. [111] encapsulate exenatide, using TPP as a cross-linking agent. Chemical modifications were performed to generate the trimethylchitosan derivative, which has a stronger positive charge, leading to a stable and viable formulation for oral use in the treatment of type-2 diabetes, as demonstrated in their pharmacokinetic and pharmacodynamic studies.

Trimethylchitosan (TMC) improves the low solubility of chitosan, particularly at physiological pH 7.4, by enhancing penetration and opening epithelial tight junctions in basic and neutral environments, thereby improving the paracellular transport of the drug molecule and reducing transepithelial electrical resistance [135–136]. In this regard, Farhadian et al. [118] manufactured trimethylchitosan NPs for encapsulating hepatitis B antigen (HbsAg) for the development of oral vaccines, coated with hydroxypropyl methylcellulose (HPMC). In vitro release studies demonstrated improved acid stability of TMC NPs, while preserving HbsAg after exposure to stomach pH, suggesting that TMC/HPMC NPs are a viable option for oral administration of the hepatitis B vaccine.

Stability is a challenge in vaccine development, especially for cancer vaccines. RNA, the key element in this process, encodes antigens responsible for humoral immunity but is inherently unstable due to its single-stranded structure and susceptibility to environmental degradation, particularly via the oral route [137]. Therefore, stabilizing elements are necessary, and NPs have been widely used because of their relatively safe, easily adjustable physical and chemical properties and high load capacity [126].

In this context, chitosan has been studied for its ability to protect nucleic acids from nuclease degradation, facilitating cellular entry through the formation of polyplexes driven by strong electrostatic interactions [138]. The positive charge of chitosan enables interactions with negatively charged nucleic acids, forming polymeric complexes. Consequently, the incorporation of genetic material can occur via encapsulation, adsorption, or electrostatic interactions, resulting in nanocapsules, micelles, and diverse morphologies and shapes, as well as varying release profiles [139].

Considering that, to reach their therapeutic target via oral administration, nucleic acids must overcome the mucosal barrier, tight junctions that block paracellular passage, epithelial cells, subepithelial tissue, and the harsh gastric and intestinal environments, a highly challenging biological context is created [140], as depicted in Figure 1. To address this challenge, and given that cysteine promotes both mucoadhesion and the opening of tight junctions, Han et al. [117] developed a trimethyl chitosan–cysteine conjugate (GTC) to formulate PNs for the co-delivery of antitumor shRNA and siRNA via oral administration. These therapeutic genes respectively induce apoptosis and inhibit angiogenesis in cancer. Following oral PN administration in tumor-bearing mice, tumor regression was observed, attributed to the synergistic effects of the two RNAs in inhibiting cell proliferation in vitro and tumor growth in vivo.

Since nucleic acids are widely utilized in cancer therapy due to their specific ability to regulate the expression of any associated gene [141] the findings of Han et al. [117] represent an effective approach for synergistic therapy via oral RNA delivery. Moreover, these findings open new possibilities for the application of chitosan conjugates with other biopolymers.

Alginate (Figure 4b) is among the most extensively studied biopolymers for forming conjugates with chitosan, as its anionic character complements chitosan’s cationic properties, resulting in a more stable nanomaterial. Additionally, alginate is water-soluble, exhibits mucoadhesive properties, and its pH sensitivity serves as a valuable tool for controlling the release of encapsulated molecules [142–143]. Thus, alginate, with carboxyl groups (COO^−^), undergoes protonation and contracts in acidic environments, while in basic environments, it swells due to the exchange of Ca^2+^ ions with Na^+^ [144].

The complementary characteristics of chitosan and alginate have been utilized to develop nanocarriers for the release of proteins and/or peptides, such as bovine lactoferrin (LF). LF is a cationic glycoprotein involved in iron transport, with immunomodulatory and anticancer activities mediated through diverse molecular mechanisms. Mahidhara et al. [145] incorporated LF into chitosan NPs coated with alginate, which solubilized the protein in an aqueous environment. Controlled release was achieved, with the nanoparticle undergoing endocytosis and transcytosis during LF transport, demonstrating the efficacy of these biopolymers in protecting the molecule from pH variations. Complementary preclinical studies validated these findings, showing that chitosan- and alginate-coated NPs enhance the bioavailability and metabolic stability of bovine LF, preventing its degradation in mouse plasma. Oral administration in mice demonstrated that the NPs crossed the mucosal barrier without damaging the molecule, exhibiting high anticancer activity against colon tumors. This effect was attributed to the improved bioavailability provided by the nanoformulation, which induced rapid tumor regression while protecting normal cells [146].

Similar results demonstrating the effectiveness of the chitosan–alginate combination were reported by Samarasinghe et al. [98], who encapsulated bovine lactoferrin in alginate–chitosan NPs for osteoarthritis treatment. These NPs were administered orally to mice, demonstrating nontoxicity and a reduction in joint inflammation, which were attributed to the physicochemical stability of the NPs in the gastrointestinal tract. Continuing their research on lactoferrin, Mahidhara et al. [112] developed chitosan-coated nanocapsules wrapped in alginate that achieved a 4.8-fold reduction in tumor size and prevented recurrence when compared to intraperitoneal injection of taxol and doxorubicin. The in vitro and in vivo findings (see Table 2) confirmed that the nanocapsules isolated the active payload, preventing enzymatic degradation, while functionalization facilitated targeted administration [32].

The ionic interaction between alginate and chitosan, forming smart, pH-sensitive polyelectrolytic nanocomposites [147], recently led Silvestre et al. [99] to encapsulate curcumin within alginate–chitosan NPs. Curcumin, a hydrophobic molecule that degrades rapidly under alkaline conditions and other environments, has significant biomedical applications [148]. In this study, NPs were incorporated into biofilms for oral administration, demonstrating controlled release and effective protection of curcumin from external factors.

The results discussed in this section highlight the potential of chitosan as a nanocarrier for innovative therapeutic platforms, providing more effective treatment options for various clinical conditions, including infectious diseases, cancer, and immunological disorders.

**4.1.2 Zein.** Zein (Figure 4c) is a vegetable protein extracted from corn, characterized by its amphiphilic nature due to a balance of hydrophobic and hydrophilic groups. Based on differences in molecular weight and solubility, zein is divided into fractions: α-, β-, γ-, and δ-zein. The α-zein fraction is the most abundant, accounting for 80% of its content, with a molecular weight ranging from 19 to 22 kDa. β-zein constitutes 10% of the total zein and is rich in methionine, while the γ- and δ-zein fractions together represent the remaining 10%. Structurally, zein predominantly consists of hydrophobic and non-polar amino acids, including glutamine, leucine, proline, and alanine [149–150].

Zein’s amino acid composition contributes to its insolubility in water, except under specific conditions, such as in the presence of high concentrations of urea, alcohol, anionic surfactants, or at high pH levels (≥11). Its hydrophobicity and solubility profile make zein an attractive nanocarrier as it physically encapsulates hydrophobic compounds, enabling sustained release. Zein is considered safe and can self-assemble into NPs with various structures, depending on the solvents and processing conditions used [151], as shown in Table 3.

The characteristics of zein enable its NPs to move slowly through the gastrointestinal tract, becoming trapped in the intestinal epithelium and persisting for more than 24 h until they are degraded or eliminated. This anti-digestibility property is significant for oral administration systems, highlighting the stability of zein-based nanocarriers [152].

Due to its ability to enhance the oral bioavailability of hydrophobic molecules, Zimath et al. [123] incorporated mometasone into zein NPs. Mometasone, a lipophilic drug with low aqueous solubility, currently lacks oral formulations. Despite its many side effects, it presents fewer diabetogenic effects compared to dexamethasone, making it a candidate of interest for oral delivery systems. In this study, the authors reported that zein NPs, measuring approximately 100 nm, promote intestinal translocation. Permeability studies indicated that the NPs effectively retain mometasone during transport across cell monolayers, resulting in slow and gradual absorption. In vitro release studies supported these findings, showing minimal release at acidic pH (1.2) and sustained, controlled release at intestinal pH (6.8). The strong and prolonged interaction of zein NPs with mucus extends the absorption time and systemic bioavailability of mometasone, suggesting their potential as a promising nanocarrier for oral administration.

Reboredo et al. [121] encapsulated insulin in zein NPs and coated them with PEG, a mucus-permeable agent, to evaluate the effects of the coating, which was applied by adsorption onto the particle surface after synthesis. This technique is frequently employed due to its simplicity and efficiency. In a rat model of diabetes, orally administered insulin-loaded NPs exhibited a hypoglycemic effect lasting 5 h, compared to 3 h for subcutaneously administered insulin. The PEG-coated NPs demonstrated greater potency.

Zein, is naturally mucoadhesive due to its intrinsic chemical properties. However, hydrophilic polymers such as PEG reduce interactions with mucosal components and enhance absorption in the intestinal epithelium by increasing mucus diffusivity, thereby improving the oral bioavailability of insulin-loaded NPs in the enterocytes, the main site of absorption. In contrast, uncoated NPs remained trapped in the mucus, positioned further from the absorptive epithelium. These findings suggest that PEG coating may be a viable strategy for improving the oral delivery of insulin by overcoming the intrinsic adhesiveness of zein, and appears to be a valuable strategy for improving the bioavailability of other biopharmaceuticals [121,153]. The role of PEG will be further explored in the section on synthetic polymers.

The interaction of zein with the intestinal mucosa, along with its ability to form stable nanoparticles with prolonged release, enhances the bioavailability of hydrophobic molecules. Therefore, based on the studies discussed in this review, zein is emerging as a viable alternative for safe and efficient drug delivery, particularly for chronic treatments that require sustained and effective release.

**4.1.3 Casein.** Casein (Figure 4d) is the most abundant protein in bovine milk, composed of four phosphoproteins: αS1-, αS2-, β-, and κ-casein [154]. These phosphoproteins are amphiphilic and form micelles through hydrophobic interactions stabilized by calcium phosphate nanoclusters, which act as bridges connecting the phosphoproteins. Casein naturally serves as a carrier of bioactive agents, reflecting its biological role in nutrient transfer from mother to offspring [155].

The versatility of biopolymers for chemical modification allows the design of casein nanocarriers to be tailored for specific therapeutic needs (Table 3). Gandhi and Roy [102] produced casein NPs loaded with doxorubicin for oral administration in the treatment of gastric cancer, requiring targeted release in the gastric region. To stabilize the nanosystem and enable release at acidic pH, they utilized excess Ca^2+^ ions, which enhanced the packaging of free casein molecules within the micellar network, precipitating as doxorubicin-loaded NPs. Approximately 90% of the drug was released at acidic pH, facilitated by proteolytic enzymes that cleave casein [156]. Additionally, the gastric environment destabilized the NPs, which exhibited a less negative zeta potential (−12.5 mV) due to the presence of excess calcium during the nanoparticle formation process.

In another scenario, Yuan et al. [104] developed casein nanocarriers with enrofloxacin, which needed to be absorbed in the intestinal region. Enrofloxacin, a broad-spectrum antimicrobial, has shown satisfactory results in animal studies for treating both extracellular and intracellular infections; however, its low solubility in water complicates its oral administration [157]. During the preparation of NPs, a casein solution adjusted to pH 12 was used to facilitate drug absorption in the intestinal region.

The release profile of the NPs was pH-dependent, with only 40% of enrofloxacin released at acidic pH after 24 h. This slow release is due to pepsin, an enzyme that cleaves peptide bonds with hydrophobic aromatic amino acids inside the casein nanoparticle, preventing drug release in the gastric environment. At basic pH, an immediate release followed by sustained release occurred due to the repulsive effect between the negative charges of enrofloxacin and casein at pH 7.4, which facilitated drug release from the nanocarrier [104,153].

The amphiphilic structure and the ability to form stable micelles enable casein to encapsulate bioactive compounds with limited solubility. The studies analyzed demonstrated that zein NPs release drugs in a controlled manner, which is pH-dependent, facilitating their adaptation to different regions of the GIT and promoting their absorption.

**4.1.4 Hyaluronic acid.** Hyaluronic acid (HA) is a natural anionic linear polysaccharide, first extracted from bovine vitreous bodies in 1934. It is composed of consecutive disaccharide units of ᴅ-glucuronic acid and *N*-acetyl-ᴅ-glucosamine linked together through 1,4- and 1,3-glycosidic bonds, as illustrated in Figure 4e [158]. Present in the body, and its polymer chain varies in size and molecular weight, which affects its biological function, determining the cell’s responsiveness to external signals and its interaction with the cellular matrix and membrane receptors [159].

HA performs physiological functions such as controlling tissue hydration and several functions related to receptor-mediated cell displacement, mitosis, and migration. The molecular weight and percentage of HA must therefore be carefully analyzed considering the therapeutic purpose and the type of carrier used [160]. It is a highly hydrophilic polymer, as disaccharides have a carboxylic group that ionizes at physiological pH, increasing its polyanionic nature. This characteristic allows metal ions to bind to the hydration envelope, increasing volume and forming loosely compacted hydrated matrices. This underpins its physiological functions such as wound healing, cell lubrication, and its rheological characteristics [161]. With the presence of various functional groups in its molecule, including glucuronic acid, carboxyl, *N*-acetyl hydroxy, and amine groups, HA can be easily conjugated and is ideal for modifying nanomaterials for oral administration [162].

At physiological pH, HA has a negative charge and functions as nanotransporter. Consequently, modifications to its surface can significantly influence its interactions with the surrounding microenvironment. This high surface potential is advantageous for maintaining the NPs in suspension, preventing aggregation or precipitation. Also, the dense negative charges cause a strong repulsive interaction in vivo between the modified nanotransporters and serum proteins that are also negatively charged, resulting in the prolongation of the plasma half-life of this nanostructure. Additionally, it creates a hydrophilic layer on the surface of the NPs, protecting them from opsonization by forming an additional energy barrier through bound water molecules, which extends their circulation time in the blood [163].

Oral absorption is increased through endocytosis since HA adheres to the mucous membranes of the gastrointestinal tract via its binding to specific receptors. This cellular uptake mechanism is influenced by the size, shape, and zeta potential of the NPs. According to [164], cells internalize spherical hyaluronic acid NPs smaller than 400 nm with a zeta potential between −10 and −20 mV. In this scenario, the pH variation in the gastrointestinal compartments affects the stability of the nanoparticle, its blood circulation time, and absorption across body membranes [165].

Wang et al. [94] (Table 3) analyzed the influence of the molecular weight of HA on the absorption of pH-responsive mucoadhesive NPs for the oral administration of insulin. The results demonstrated that NPs manufactured with high molecular weight HA exhibit greater protective capacity and a more pronounced prolonged release effect, extending the NPs’ permanence at the absorption site and enhancing the efficacy and absorption of insulin.

Due to the properties of HA and its ability to be functionalized with small molecules, Agboluaje et al. [96] developed hyaluronic acid NPs functionalized with bile acids, deoxycholic acid (DOCA) and taurocholic acid (TCA) to improve the oral absorption of deferoxamine (DFO). In this study, sonication was used within a broader preparation method that involves chemical conjugation and oxidation for the formation of NPs, aiming at the dispersion of aggregates and standardization of nanoparticle size.

DFO is an iron chelator used in patients with hemopathies, such as sickle cell anemia [166]. Bile acids were also evaluated to enhance oral absorption, and polymeric conjugates (HA-DFO, DOCA-HA-DFO, and TCA-HA-DFO) were synthesized. In vitro analyses for information on cytotoxicity, absorption, and chelation efficacy were performed, demonstrating that the oral absorption of the NPs increased as more TCA molecules were conjugated to hyaluronic acid. This likely occurs due to the increased hydrophilic nature, which facilitates their movement through the aqueous environment.

Considering the studies discussed in this review, HA’s high hydrophobicity and surface modulation capacity make it a promising biomaterial for oral nanocarriers, enhancing drug delivery and promoting an effective and controlled therapeutic approach.

#### 4.2 Synthetic polymers and polymeric nanoparticle systems for oral use

Synthetic polymers are the most widely used polymeric nanocarriers due to their ability to control physicochemical properties, biodegradation, and chemical functionalization. Among the most common synthetic polymers, polyesters exhibit distinct characteristics based on their chemical properties, such as prolonged release, cell permeability, and ease of chemical conjugation. The best-known representatives include PLGA, PCL, and PEG [167].

Another class of synthetic polymers is the methacrylates, whose most widely used component is Eudragit^®^ (RS, RL, S, E), available in different types depending on its solubility profile. Eudragit is widely used for its pH sensitivity, enabling the intestinal release of proteins and vaccines following oral administration. Polymers derived from maleic anhydride represent a newer synthetic option for oral NPs and are also being used for antigen delivery. Poly(methylvinyl ether-*co*-maleic anhydride) (PVM/MA), commercially known as Gantrez^®^, is the main representative of this class. Polymers such as polyhydroxybutyrate (PHB) and polyvinyl alcohol (PVA) are also employed in nanoformulations [168–169].

**Figure 5:**
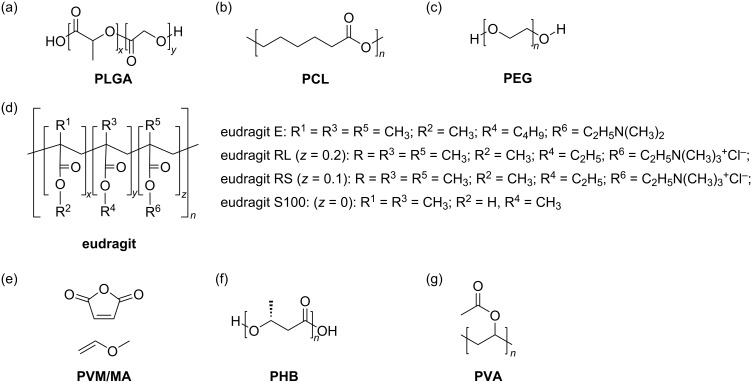
Chemical structures of the most commonly used synthetic polymers in polymeric nanoparticles (PNs) for oral administration.

Figure 5 illustrates the chemical structures of these synthetic polymers, which, as the most widely used oral nanocarriers, will be discussed in this review, highlighting their physicochemical characteristics and current applications in the encapsulation of drugs, biological molecules, genes, and other substances.

As demonstrated in Section 4.1, oral NPs, although extensively studied for conventional drugs, represent an emerging and highly promising field. Consequently, synthetic polymers are also being investigated to address challenges related to the oral delivery of nucleic acids. This is due to their ability to encapsulate, adsorb, or conjugate various substances to their core or surface, thereby providing protection and controlled release. Their mucoadhesive, stimulus-responsive, or ligand-specific properties facilitate targeted oral gene delivery [170].

The following sections will present examples of the application of synthetic polymers in the delivery of nucleic acids, antigens, vaccines, and conventional drugs.

**4.2.1 Poly(lactic-*co*-glycolic acid).** PLGA is one of the most widely used and promising materials as a nanocarrier in drug delivery, as it promotes controlled release and regulates biodegradation. It is capable of entrapping most therapeutic products by manufacturing particles of varying sizes and shapes. The molecular weight, lactic-to-glycolic acid ratio, and concentration of the encapsulated active are parameters that adjust drug release. Another advantage of PLGA is its approval by the FDA for oral, topical, and intravenous use in humans, due to its biocompatibility and safety [171].

PLGA (Figure 5a) is a linear aliphatic polyester copolymer composed of lactic acid and glycolic acid, which can exhibit varying proportions depending on the manufacturing method [172]. Although it can be synthesized through various methods, the primary ones are polycondensation and ring-opening polymerization reactions, producing PLGA with low and high molecular weights, respectively. Reaction conditions influence the polymer’s physicochemical properties, as the monomer sequence affects its degradation rate and other physicochemical characteristics. The varying monomer proportions impact amphipathic properties, including polymer emulsification capacity. An increased glycolic acid content enhances the polymer’s hydrophilicity, resulting in faster degradation rates. Conversely, PLGA demonstrates greater resistance to hydrolysis when the terminal group is an ester, which occurs with a higher lactic acid proportion [173–174].

PLGA biodegrades through the cleavage of polymer chains via non-enzymatic hydrolysis of ester bonds or by mass erosion, releasing lactic and glycolic acids. As natural metabolites, these acids are eliminated through the Krebs cycle in the form of carbon dioxide and water. Consequently, PLGA undergoes complete degradation within a short period, minimizing the risk of adverse reactions [175].

Anwer et al. [176] prepared PLGA nanoparticles loaded with rivaroxaban (Table 4) with the aim of achieving sustained release to reduce the need for fasting doses. Rivaroxaban is an anticoagulant with low solubility, available in doses of 10, 15, and 20 mg, and its bioavailability is influenced by food intake. Release and pharmacokinetic studies were conducted comparing the nanoparticle formulation (RXB-PLGA-NP) with the commercial formulation (Xarelto^®^). The nanoparticle exhibited improved and sustained release of rivaroxaban over 48 h (97.64%) compared to Xarelto^®^ (68.45%). Additionally, the relative bioavailability of the NPs during fasting (256%) and feeding (216%) were similar, as were the maximum absorption levels during fasting (553 ± 157 ng/mL, 4196 ± 546 ng·h/mL) and feeding (566 ± 40 ng/mL, 3589 ± 138 ng·h/mL). The authors attributed the sustained release pattern and increased bioavailability of the drug in the absence of food effects to the presence of the PLGA polymeric matrix and the nano-sized particles.

**Table 4:** Studies with synthetic polymers for oral nanoparticles.

Polymer	Drug and BCS	Therapeutic class	Preparation technique	Ref.
Objective Results (Release, In vitro test, In vivo test)				

Methacrylic acid-ethyl acrylate	glibenclamide (II)	hypoglycemic agent	solvent displacement	[177]
Objective: Develop, characterize, optimize and evaluate in vivo PNs of a methacrylic acid-ethyl acrylate copolymer loaded with glibenclamide in diabetic rats as an alternative for the treatment of type-2 diabetes. Release: Rapid release at acidic and neutral pH and sustained release at pH 7.4 lasting 50 h. In vitro test: – In vivo test: 0.9 mg of glibenclamide encapsulated in NPs demonstrated better efficacy in glycemic control in diabetic rats, compared to the control group.				

Eudragit^®^ L100	insulin (N/A^a^)	hypoglycemic agent	own method	[178]
Objective: Synthesize and evaluate insulin-loaded PNs (PNs) composed of thiolated Eudragit ® L100 (Eul-cys) and reduced glutathione (GSH) as potential nanocarriers for oral administration of insulin. Release: Insulin release was faster from PNs without GSH than from Eul-cys/GSH PNs at pH 7.4, suggesting that GSH may influence particle release. In vitro test: Insulin is transported by a paracellular process. Eudragit ® PNs with sodium caprate showed a greater increase in permeation compared to reduced glutathione. In vivo test: –				

Eudragit^®^ E100	naringenin (N/A^a^)	antineoplastic	emulsificatio; solvent difusion/evaporation	[179]
Objective: Prepare Eudragit ® E 100 PNs loaded with naringenin. Release: Release via a Fickian diffusion mechanism. In vitro test: PNs have increased cytotoxicity compared to free naringenin. In vivo test: Significantly improved oral bioavailability.				

Eudragit^®^ RS, Eudragit^®^ E	triclabendazole (II/IV)	antiparasitic	nanoprecipitation	[180]
Objective: Optimize pH-sensitive Eudragit® PNs loaded with triclabendazole using experimental design for an immediate-release oral system. Release: Enhanced release in the presence of Tween 80 with improved drug wettability and dissolution. In vitro test: – In vivo test: –				

Eudragit^®^ RL 100	ketoprofen (II)	anti-inflammatory	emulsification	[181]
Objective: Develop a ketoprofen-loaded Pickering nanoemulsion using PNs. Release: Slower release at pH 1.2 and increased at pH 6.8. 15% was released within 2 h, 70% after 5 h and 96% within 9 h. In vitro test: – In vivo test: –				

Eudragit^®^ RS, Eudragit^®^ S100	–	–	emulsification/solvent diffusion	[182]
Objective: Investigate the in vivo distribution in mice after oral administration of two formulations: unencapsulated PNS suspension and PNS-containing microcapsules. Release: PNs released 13% of their fluorescent dye (Cy5) payload in simulated gastric, intestinal and colonic fluids. In vitro test: HT29 cells showed higher uptake than NIH/3T3 cells. Efficient non-specific uptake of PNs by cancer and normal cells. In vivo test: PNs cleared faster from the mouse gastrointestinal tract than fluorescent PNs. Minimal uptake of NPs.				

Gantrez^®^	–	–	solvent displacement	[183]
Objective: Prepare thiamine-coated PNs using two distinct preparative processes, and to evaluate and compare their properties and mucus permeation behavior in vivo. Release: – In vitro test: – In vivo test: Oral administration of PNs revealed their localization in the stomach and small intestine. The thiamine coating of PNs appeared to move faster through the digestive tract compared to those without thiamine. No accumulation was observed in the liver or lungs.				

Gantrez^®^	–	–	solvent displacement	[184]
Objective: Explore in vitro genotoxicity of poly(anhydride) PNs and Gantrez's after 24 h using the alkaline comet assay and the mouse lymphoma test (MLA) in L5178Y TK+/− cells. Release: – In vitro test: GN-NP and GN-MA-NP did not affect cell viability. GN-NP and GN-MA-NP were cytotoxic to ML cells. In vivo test: The PNs did not induce DNA strand breakage or oxidative damage and there was no induction of significant or biologically relevant genetic mutations. Absence of genotoxicity.				

Gantrez^®^; PEG	–	–	solvent displacement	[185]
Objective: Investigate the mucus permeation of PEG-coated PNs, derived from the Gantrez® AN copolymer, after oral administration in rats. Release: – In vitro test: – In vivo test: PEGylated PNs remained in the stomach and small intestine after oral administration to animals. PNs without PEG coating showed a greater ability to interact with the stomach mucosa.				

Gantrez^®^; poly(ethylene sebacate)	rifampicin (II)	tuberculostatic	nanoprecipitation	[186]
Objective: Develop PNs of Gantrez and poly(ethylene sebacate) with rifampicin (RIF) and compare pharmacokinetics and biodistribution. Release: – In vitro test: Polyethylene sebacate PNs showed greater uptake by macrophages than Gantrez PNs, with internalization being mediated by the folate receptor. In vivo test: PNs demonstrated significantly higher *C*_max_, AUC, delayed *T*_max_ and sustained release compared to free drug. Plasma concentration of RIF with PNs was considerably higher than its free form over 24 h.				

Hydroxypropyl methylcellulose (HPMC)	docetaxel (IV)	anti-inflammatory	solvent evaporation	[187]
Objective: Develop oral delivery systems for PNs based on HPMC, chitosan (HTC) and polyethylene glycol ᴅ-α-tocopherol succinate (TPGS) for controlled release of docetaxel (D). Release: After 30 days, PNs cross-linked with PVA (86%), PNs in which the drug was loaded by incubation (56%) and PNs formed by solvent evaporation (75%). In vitro test: PNs with the drug showed greater suppression of cell viability of the MDA-MB-231 cancer cell line than the free drug. PVA-cross-linked PNs showed better cellular uptake and increased permeability by 3.5 times compared to the free drug. In vivo test: –				

mPEG-PCL	donepezil (I)	cholinesterase inhibitor	nanoprecipitation	[188]
Objective: Develop brain-targeted lipoprotein-modified PNs. Release: Biphasic release profile: an initial burst followed by a sustained release. Korsmeyer–Peppas model, with *n* < 0.43, indicates a Fickian diffusion mechanism. In vitro test: Cell viability >85–90%. Absence of toxicity. Significantly higher uptake in U87 cells (85.96 ± 1.86) compared to pure drug (45.94 ± 2.65). In vivo test: Nanoparticle conjugated with lipoprotein (D3-PNs) presented a larger area under the curve compared to the other formulations and the free drug.				

mPEG-PCL	indinavir (IV)	immunosuppressant	Emulsification/Solvent evaporation	[189]
Objective: Design, prepare and evaluate optimized polymer-based PNs loaded with indinavir as potential delivery systems for oral drug administration. Release: Biphasic release pattern, with initial burst followed by sustained release. Release rate is pH-dependent and influenced by drug-polymer interactions. The Korsmeyer–Peppas model best describes the release kinetics. In vitro test: High permeability in CaCo-2 cells and significant increase (*p* < 0.05) in PNs uptake through the cell monolayer compared to free drug. In vivo test: Nanoparticulate system presented an area under the curve 5.32 times greater than the indinavir solution.				

mPEG-PCL	thymoquinone (N/A^a^)	antineoplastic	nanoprecipitation	[190]
Objective: Develop and evaluate a mPEG-PCL-based polymeric nanoformulation for delivery of thymoquinone as a potential anticancer nanomedicine. Release: F1 and F2 NPs showed biphasic release, with F1 releasing 86–90% in 8 h versus 76% for F2 at pH 7.4. In vitro test: Thymoquinone had stronger antiproliferative activity, but NPs showed higher anticancer selectivity (64 vs 6.7). In vivo test: The area under the curve of formulation F2 was 1.58 times greater than the area under the curve of the drug in suspension.				

PAA (poly(acrylic acid)); chitosan	compost FD (N/A^a^)	antiparasitic	nanoprecipitation	[191]
Objective: Investigate the effect of thiolate anionic poly(acrylic acid) and cationic chitosan used in free soluble form and as a nanoparticulate system on the absorption of the hydrophilic compound FD. Release: – In vitro test: There was no induction of severe toxicity in Caco-2 and MTT cells. Cell viability of 80% and mitochondrial activity of 85%. Thiolate formulations increased permeation 5.3-fold. Decreased transepithelial electrical resistance of the cell layer. In vivo test: –				

PCL	insulin (N/A^a^)	hypoglycemic agent	double emulsion/solvent evaporation	[192]
Objective: Develop a pH-sensitive polymeric system for oral insulin delivery based on a PCL core and chitosan (CS) and alginate (ALG) shells. Release: Insulin released by PCL PNs increased from 36% to 81% as added Pluronic127 increased from 0.1% to 5% w/v. In vitro test: – In vivo test: –				

PCL	α-tocopherol (N/A^a^)	anti-inflammatory	nanoprecipitation	[193]
Objective: Examine the oral effects of α-tocopherol-loaded polycaprolactone PNs on joint inflammation and oxidative stress in plasma, liver and brain of middle-aged rats. Release: – In vitro test: – In vivo test: Administration of α-tocopherol did not affect the activities of aspartate and alanine aminotransferase in plasma and increased their content in the liver and brain of arthritic rats.				

PCL	ivermectin (II)	antiparasitic	nanoprecipitation	[194]
Objective: Develop a new ivermectin formulation based on PCL nanocapsules formulated with pumpkin seed oil (PSO). Release: – In vitro test: Nanocapsules mitigated ivermectin toxicity in L929 fibroblasts and J774 macrophages with maintenance of cell viability and anthelmintic activity against *Strongyloides venezuelensis*. In vivo test: –				

PEG; PCL	nucleic acid (N/A^a^)	anti-inflammatory	emulsification ,solvent evaporation	[195]
Objective: Develop and evaluate a novel nanodelivery system of PEG-PCL PNs with encapsulated nucleic acid complexes - mannosylated PEI (Man-PEI) for intestinal delivery. Release: – In vitro test: Cellular internalization of the nanoparticle occurs by mannose receptor-mediated endocytosis with minimal toxicity in SW480 and HCT-15 cells. In vivo test: PEG-PCL PNs transfected the plasmid expressing green fluorescence protein into mouse intestinal cells.				

PEG; PLGA	bavaquinine (N/A^a^)	N/A^a^	solvent evaporation	[196]
Objective: Produce oral PNs to treat asthma by improving the aqueous solubility of bavaquinine. Release: – In vitro test: Negligible cytotoxicity in vitro and PNs taken up by Caco2 cells. In vivo test: PNs accumulated significantly in the lungs of asthmatic mice, but not in healthy mice, suggesting lung uptake in the asthma model.				

PEG-poly(anhydride)	insulin (N/A^a^)	hypoglycemic agent	desolvation	[197]
Objective: Evaluate the potential of mucus-permeable PNs based on coating zein PNs with a polymeric conjugate containing PEG as carriers for oral insulin delivery. Release: – In vitro test: – In vivo test: Oral administration of insulin in *C. elegans* reduced intracellular glucose and fat content, with nanoencapsulated insulin having a more significant effect than free insulin.				

PLGA	cyclosporine (II)	immunosuppressant	emulsification, solvent evaporation	[198]
Objective: Evaluate and compare the relative bioavailability of PLGA PNs with two types of lipids nanocarriers. Release: PLGA PNs release cyclosporine by erosion over days, resulting in minimal drug release within 12 h. In vitro test: – In vivo test: Oral bioavailability: PLGA nanoparticle (22.7%), self-emulsifying systems (73.6%) and lipid PNs (118.8%).				

PLGA	–	–	emulsification, solvent evaporation	[199]
Objective: Assess *Drosophila* larvae as a low-cost model for real-time drug screening to enhance oral PNS formulations. Release: – In vitro test: No significant reduction in cell viability was observed at increasing concentrations of coated and uncoated PNs. In vivo test: Coated PNs have a long retention time in the intestine with increased mucoadhesive capacity, and uncoated PNs permeate smoothly through the intestine.				

PLGA	insulin (N/A^a^)	hypoglycemic agent	solvent diffusion	[200]
Objective: Propose a new concept of microcapsules composed of pH-sensitive poly(lactide-*co*-glycolide) PNs for oral administration of insulin. Release: Microcapsules composed of PLGA NPs showed slower and more sustained insulin release than PNs at acidic pH. Both released more than 90% of insulin at pH 7.4 in 24 h. In vitro test: – In vivo test: Insulin released in 12 h maintained its bioactivity during the preparation process; The enteric nature of the polymer particles helped to improve the in vivo response for 24 h from a single application; Relative bioavailability of 15.6% for the composite microcapsules and less than 0.5% for free insulin.				

PLGA	etoposide (N/A^a^)	antineoplastic	emulsification, solvent evaporation	[201]
Objective: Develop and evaluate dual-loaded PNs containing etoposide and quercetin to increase oral bioavailability through inhibition of P-glycoprotein. Release: 68.9 ± 3.2% of etoposide was released in 48 h. In vitro test: Reduction in IC50 values for all etoposide-loaded PNs and deeper intestinal permeation for etoposide + quercetin dual-loaded PNs. In vivo test: Etoposide + quercetin nanoformulation significantly increased the area under the curve compared to the free etoposide formulation. Etoposide nanoformulation alone improved the area under the curve compared to the traditional formulation.				

PLGA	nuciferine (N/A^a^)	N/A^a^	double emulsion	[202]
Objective: To develop nuciferine-loaded PLGA PNs using a solid/oil/water emulsion technique. Release: Rapid release in gastric fluids and sustained in intestinal fluids. In vitro test: Demonstrated the role of PNs in alleviating lipogenesis. Cell viability >90%. Absence of cytotoxicity and apparent apoptosis. In vivo test: PLGA nanoparticle with nuciferine provided 3.39 times greater bioavailability than traditional oral administration.				

PLGA	resveratrol (II)	antineoplastic	solvent diffusion	[203]
Objective: To develop and evaluate galactosylated PLGA nanoparticle (GNPs) for oral administration of resveratrol (RES), to increase oral bioavailability and anti-inflammatory effect in vitro. Release: Galactosylated and normal PNs showed slow release, with less than 43% of the drug released in 8 h. There was no difference in release between the two formulations. In vitro test: Galactosylated PNs increased drug permeability in all intestinal segments. Internalization occurs by clathrin-mediated endocytosis via ligand–receptor/transporter interaction. In vivo test: The area under the curve of the galactosylated formulation was 3.35 times greater than that of the resveratrol suspension. Nanoformulation without galactose showed a 1.65-fold increase in relation to the RES suspension.				

PLGA	mycophenolate mofetil (II)	immunosuppressant	emulsification/solvent evaporation	[204]
Objective: Develop a sustained-release oral formulation of mycophenolate mofetil using chitosan-coated polylactic acid (PLA) and PLGA PNs, evaluating the role of polymer molecular weight in encapsulation efficiency (EE) and in vitro nanoparticle release. Release: PLA and PLGA PNs with a biphasic release pattern, with an initial burst followed by a sustained release. Release rate influenced by molecular weight and polymer composition. In vitro test: – In vivo test: –				

PLGA	curcumin (IV)	antineoplastic	emulsification/solvent evaporation	[205]
Objective: Systematically study the penetration properties of PLGA/Pluronic F-127 (PF127) PNs in colonic mucus and their application in the oral treatment of ulcerative colitis. Release: – In vitro test: PF127 enhanced mucus penetration and PNs internalization without toxicity in RAW 264.7 macrophages, reducing TNF-α levels in vitro. In vivo test: Nanoparticle containing PF127 showed increased penetration and mucus accumulation in inflamed colon tissues.				

PLGA	imatinib (I)	antineoplastic	solvent diffusion	[206]
Objective: Investigate the effect of galactose ligand on oral absorption of imatinib facilitated by PNs. Release: Significantly slower and sustained drug release in galactosylated PNs compared with imatinib solution and suspensions. In vitro test: Galactosylated PNs exhibit greater intestinal permeability and present multiple transport mechanisms to enterocytes. In vivo test: The PNs without galactosylation and galactosylated PNs presented, respectively, an area under the curve of 1.15 and 1.51 times greater than the suspensions of the free drug.				

PLGA	curcumin (IV)	antineoplastic	emulsification, solvent evaporation	[207]
Objective: Develop and evaluate a novel type of nanoparticle (NP) loaded with curcumin (CUR) functionalized by porous Pluronic F127 (porous PF127-NPs) for targeted delivery and controlled release in colitis tissues. Release: Biphasic release pattern, with an initial burst followed by sustained release. Release rate influenced by pH and porosity. Faster drug release in acidic conditions. In vitro test: Non-toxic PNs in RAW 264.7 macrophages and CT-26 cells. Non-porous PNs have a higher density than porous ones, tending to sediment away from the cells more quickly during treatment. Porous PF127-NPs have a greater capacity to inhibit pro-inflammatory cytokines. In vivo test: PF127-NPs reduced myeloperoxidase activity, spleen weight, and pro-inflammatory cytokine levels in mice with induced colitis, suggesting their potential as anti-inflammatory agents.				

PLGA	peptide (N/A^a^)	antineoplastic	double emulsion	[208]
Objective: Develop targeted PNs to boost L-cell stimulation for improved oral peptide delivery in incretin-based diabetes treatment, comparing lipid and PNs in vitro and in vivo. Release: – In vitro test: PNs do not increase GLP-1 secretion in in vitro assays. In vivo test: –				

PLGA	rivaroxaban (II)	anticoagulant	emulsification/solvent evaporation	[176]
Objective: Develop and evaluate rivaroxaban-loaded PLGA nanoparticle (RXB-PLGA-NPs) for oral administration, with the aim of reducing dosing frequency in the fasting state. Release: RXB-PLGA-NPs showed sustained release of 97.64% after 48 h compared to the commercial tablet Xarelto® (68.45%). In vitro test: – In vivo test: Nanoparticulate formulation showed absorption 2.41 times greater than the commercial drug when administered with food and 1.22 times greater on an empty stomach.				

PLGA	insulin (N/A^a^)	hypoglycemic agent	double emulsion/solvent evaporation	[209]
Objective: Investigate and mitigate mutual interference between surface ligands in multifunctional PNs to optimize drug delivery efficacy and develop ligand-switchable PNs. Release: – In vitro test: PNs showed mucus penetration capacity and no negative impact on cytotoxicity was found; pH-dependent cellular uptake. Cellular internalization via clathrin-dependent endocytosis, with lysosomal escape protecting insulin from degradation. In vivo test: The area under the curve of the nanoparticle was 19.09 times greater than that of orally administered free insulin.				

PLGA	antigen	–	double emulsion/solvent evaporation	[16]
Objective: Develop surface-modified PLGA nanocarriers protecting a protein-based antigen in the stomach to enable potential antigen delivery at target intestinal sites. Release: Uncoated PNs: 30% at 6 h; coated PNs: 5–15%. Cumulative release after 96 h was higher for uncoated (78%) than for coated PNs (53–48%). In vitro test: Nontoxicity with cell viability of 85% while optimized formulations were 74%.; In vitro mucin binding rate 33% (PEG coated), 48% (alginate coated), 17% (uncoated). In vivo test: –				

PLGA; PEG	RNAm (N/A^a^)	–	double emulsion	[210]
Objective: Develop a method to modify the surface charge of siRNA-encapsulating PNs and assess its impact on oral siRNA efficacy for treating disorders. Release: – In vitro test: – In vivo test: Orally administered PNs with different surface charges showed therapeutic efficacy in silencing TNF-α in a murine model of colitis.				

PLGA; PEG	insulin (N/A^a^)	hypoglycemic agent	polyelectrolytic complexation	[211]
Objective: Develop and evaluate a new drug nanocarrier for oral delivery of peptides. Release: Protected from premature release into intestinal fluids by the surrounding polymer. Insulin release at pH 4.0. In vitro test: PNs bypassed the mucus barrier, reduced toxicity of insulin enhancers, and did not affect tight junctions, with limited transepithelial transport and low cellular internalization. In vivo test: The lack of correlation between in vitro and in vivo data suggests that PNs may form a depot reservoir in the intestinal epithelium, leading to limited insulin transport and a mild systemic effect.				

Cationic hyper- branched poly(amine ester) (HPAE)	sodium arsenite (NaAsO_2_) and TH287 (N/A^a^)	N/A^a^	nanoprecipitation	[212]
Objective: Develop pH-responsive nanodelivery system based on cationic hyperbranched poly(amine-ester) (HPAE) for codelivery of sodium arsenite (NaAsO_2_) and TH287 for novel cancer therapy. Release: HPAE PNs (NaAsO_2_ + TH287) exhibited sustained release of NaAsO_2_ and TH287 in phosphate buffered saline (PBS), while rapid release was observed in acetate buffer. In vitro test: Effective inhibition of tumor cell proliferation with synergistic effect of NaAsO_2_ and TH287 that entered cancer cells and released the actives in response to acidic intracellular environments. In vivo test: –				

Polystyrene	–	–	film stretching	[213]
Objective: Investigate the role of nanoparticle size in oral drug delivery, using PNs of different sizes and shapes. Release: – In vitro test: Cellular uptake was higher in Caco-2 cells than in Caco-2/HT-29 cells. In vivo test: –				

PEGylated polyhydroxy- butyrate conjugated with deoxycholic acid	insulin (N/A^a^)	hypoglycemic agent	solvent evaporation	[214]
Objective: Develop copolymeric PNs of PEGylated polyhydroxybutyrate conjugated with deoxycholic acid loaded with insulin and perform in vitro and in vivo tests. Release: Enteric-coated PNs released insulin very slowly under acidic conditions, but released about 7% at intestinal pH after 24 h. In vitro test: 8.5-fold increase in internalization of NPs that were rapidly taken up by HCT-116 human colon cancer cells via endocytosis. In vivo test: Oral Eudragit^®^ S-100 PNs released insulin slowly, peaking at 24 h with an area under the curve of 36.95 ± 6.54 μIU h/mL.				

PVM/MA	glibenclamide (II)	hypoglycemic agent	desolvation	[215]
Objective: Design, optimize and characterize glibenclamine (GB)-loaded nanocarriers based on the combination of polyvinylmethyl ether (PVM) and maltodextrin (MA) with cyclodextrins (CDs) for oral administration purposes. Release: Slow release in acidic medium, but rapid in basic medium. Coencapsulation with cyclodextrins accelerated the release of GB. In vitro test: – In vivo test: –				

Eudragit® L 100	enoxaparin (N/A^a^)	anticoagulant	polyelectrolyte complexation	[216]
Objective: Develop Eudragit ® coated PNs loaded with Enoxaparin (Enox) through an environmentally friendly method for oral administration. Release: Cumulative release of Enox for all nanoformulations (F1, F2 and F3) was slow and remained below 10%, with F2 showing the highest release of 8.5%. In vitro test: – In vivo test: –				

TMC *N*-(2-hydroxypropyl) methacrylamide (HPMA)	insulin (N/A^a^)	hypoglycemic agent	polyelectrolyte complexation	[217]
Objective: Develop and evaluate a new self-assembled NP platform for oral insulin delivery. Release: Encapsulated insulin was released in a sustained manner at pH 6.0 and 7.4, with a slower release rate. Release was faster at pH 2. In vitro test: PNs exhibited free Brownian motion and high permeability in mucus via transepithelial transport through the paracellular pathway. pHPMA coating improved the ability to open tight junctions between mucus-secreting epithelial cells. In vivo test: Reduced blood glucose levels, with HPMA coated PNs having a more significant hypoglycemic effect.				

^a^N/A: Refers to substances to which the biopharmaceutical classification cannot be applied because they are biological or similar molecules.

This polymeric matrix exhibits a three-phase release profile, characteristic of PLGA particles, although two-phase or single-phase patterns have also been documented [24]. The process begins with an initial burst caused by weakly encapsulated cargo or cargo adhered to the particle surface, followed by a slow and sustained release mediated by the progressive hydrolysis of the polymer matrix. The third phase involves the final release of the remaining substance due to particle erosion [218].

In 2017, Liu et al. [202] discussed this release behavior of PLGA as part of their efforts to enhance the oral administration of nuciferine, a drug with limited clinical application despite its antihyperlipidemic activity, due to its low bioavailability. These results reported are promising, as they utilized simulated gastric and intestinal fluids to study the release behavior of PLGA NPs. Their findings showed a rapid release of nuciferine within the first 12 h in both gastric and intestinal fluids, attributed to the drug being weakly bound or located near the particle surface. This was followed by a slower release in gastric fluids and a sustained, prolonged release in intestinal fluids. According to the authors, the acidic environment protonated the carboxyl groups of PLGA, leading to the aggregation of the NPs into a stable structure, thereby limiting drug release. Conversely, the basic environment facilitated the release of nuciferine by increasing water absorption and penetration into the particle core.

Structural modifications can be made to PLGA nanoformulations to enhance stability, release profiles, drug targeting, and particle circulation half-life. The most commonly used agents for such modifications are PEG and poloxamers. PEGylation, as it is termed, enhances immune functionality by leveraging its hydrophilic nature to prevent particle clearance by the reticuloendothelial system. Additionally, it improves blood interactions and increases mucus adhesion in gut-associated lymphoid tissue [219].

Wang et al. [196] prepared PEG-coated PLGA NPs for the oral administration of bavachin (NP-PLGA-PEG-Bav) and administered them to asthmatic mice. The study identified greater drug accumulation in the lung region, leading to reduced inflammation and clinical improvement compared to the free drug and uncoated NPs. PEG enhanced the molecule’s targeting to the target cells and improved tissue penetration.

Iqbal et al. [210] encapsulated siRNA in PEG-PLGA nanoparticles with amine groups on the surface for charge adjustment as the delivery of this molecule relies on cationic electrostatic interactions. siRNA plays a crucial role in reducing inflammation in cases of ulcerative colitis. Nucleic acids, however, are challenging to administer orally due to their degradation throughout the GIT, necessitating the use of PEG in the NPs. In vivo studies demonstrated the structural stability and integrity of siRNA following enzymatic degradation, with accumulation in the inflamed colon and promotion of its therapeutic action.

The results observed with PEGylation of PLGA systems can be attributed to the steric repulsion and van der Waals forces provided by the hydrated barriers of polyethylene glycol on the particle surface. The flexibility of PEG chains allows for free rotation of polymeric units, forming a hydrophilic crown around the NPs. This prevents interaction with immunological markers, increases circulation time in the body, and enhances the delivery of the active ingredient to the therapeutic target [220].

PLGA nanoparticles possess a negative surface charge, which can be converted to positive by modification with cationic polymers. This modification enhances interactions with the cell membrane, facilitating the absorption of NPs by macrophages and dendritic cells. Additionally, charge inversion can induce immune responses and enable cross-presentation of antigens [221]. Mohammed et al. [204] demonstrated the benefits of this positive charge by coating PLGA NPs loaded with mycophenolate mofetil with chitosan, a cationic polymer. The resulting zeta potential greater than zero enabled mucoadhesiveness, with minimal release in simulated gastric fluid and complete release over a prolonged period in intestinal fluid.

Considering the advantages of positive charge in PNs absorption, Amin et al. [16] chemically modified the surface of PLGA NPs to enable oral vaccine delivery. To identify the most suitable polymer for functionalization, they tested alginate, polyethylene glycol, and Eudragit, all of which were incorporated after nanoparticle formation. Alginate-coated NPs exhibited greater mucin binding and sustained protein release, making them the ideal formulation for delivering protein-based antigens to trigger a mucosal immune response. These findings demonstrate the ability of polymeric nanocarriers to encapsulate antigens, protecting them against gastrointestinal degradation and enhancing their uptake by M cells in Peyer’s patches [222].

Poloxamers (also known as Pluronic^®^) have been used as a coating on PLGA nanoparticles due to their amphiphilic characteristics, which favor interactions with hydrophobic surfaces and biological membranes [223]. Poloxamers are copolymers consisting of two hydrophilic blocks of PEG separated by a hydrophobic block of poly(propylene oxide) (PPO). They are characterized by their ability to form various polymorphs, ranging from gel to solid states, depending on the molar mass ratios between the blocks. Their commercial nomenclature includes a letter indicating the morphism (L - liquid, P - paste, F - flakes) accompanied by digits (two or three) that correspond to structural parameters [224].

Considering the properties of poloxamers Zhou et al. [205] produced PLGA nanoparticles loaded with curcumin, incorporanting Pluronic F-127 during the nanoparticle formation process. The polaxamer spontaneously arranged at the nanoparticle interface, resulting in the formation of a coating layer. This functionalization enhanced mucus penetration and increased cellular uptake efficiency in macrophages, with the coated particles exhibiting greater anti-inflammatory activity and therapeutic efficacy. Among the various pharmacological actions of curcumin, its anti-inflammatory activity and ability to reduce cell infiltration have led to its application for ulcerative colitis, an inflammatory disease affecting the colonic mucosa [225]. Despite its potential, curcumin’s biopharmaceutical limitations restrict its use, making the results reported particularly promising in the field of nanotechnology.

As discussed in this review and other studies [226–228], PLGA nanoparticles are the most promising nanocarriers due to their ability to encapsulate a wide variety of pharmaceutical actives, providing controlled release and biodegradation. Modifications to the PLGA surface improving its properties, enhancing stability, reducing enzymatic degradation, modulating interaction with target cells, and positively influencing nanoparticle absorption and immune response [229]. Therefore, PLGA can revolutionize oral treatments and offer new perspectives for various therapies.

**4.2.2 Polycaprolactone.** PCL (Figure 5b) is an aliphatic, linear, semicrystalline, and hydrophobic polyester polymer. Its application in nanostructured systems is attributed to its slow biodegradation, biocompatibility, low toxicity, and thermal stability. Additionally, the ease of surface modifications enhances its adaptability to various configurations. It is a product approved by the FDA for human use [230].

PCL is synthesized through the ROP of the ε-caprolactone monomer, which can proceed via cationic, anionic, or monomer-activated and coordination–insertion mechanisms. The ROP approach used influences molecular weight distribution, end-group composition, and the chemical structure of the copolymers [231].

The advent of faster-degrading polymers such as PLGA and PLA limited the use of PCL for many years. This was due to the prevailing belief in the scientific literature that its low degradation rate would hinder its application in NPs. However, with reduced particle size, the surface area increases, leading to an accelerated degradation rate. This occurs because the enzymatic effect is surface-driven, as diffusion within the hydrophobic polymer matrix is limiting. Consequently, the degradation process involves enzymatic adsorption followed by hydrolysis, which facilitates polymer breakdown. Therefore, a larger surface area correlates with higher degradation rates [232].

Considering the slow degradation of PCL, its use in oral NPs has been attracting interest, particularly for sustained drug administration. In this context, the PCL release profile is biphasic, beginning with water diffusion through amorphous regions, followed by the hydrolytic cleavage of ester bonds. This process results in mass loss and increased crystallinity due to the formation of low-molecular-weight oligocaprolactone (OCL), which undergoes phagocytosis and is subsequently cleaved by esterases. The second phase involves the hydrolytic degradation of these crystalline domains, with chain cleavage occurring either at the final position or randomly, depending on high or low temperatures, respectively, and is autocatalyzed by the carboxylic acids formed during the reaction. Oxalate ester bonds are broken by hydrogen peroxide, producing a reactive intermediate that instantly decomposes into carbon dioxide [233]. Table 4 describes studies using PCL-based NPs.

Insulin instability in the gastrointestinal tract is a significant challenge for oral administration. To address this Kalaycioglu et al. [192] protected insulin-loaded PCL NPs by coating them with alginate and chitosan. Additionally, Pluronic F-127 was incorporated into the hydrophobic PCL matrix to enhance water permeability. Alginate was selected for its pH sensitivity, while cationic chitosan was used to neutralize the anionic alginate and facilitate permeation through the gastrointestinal tract. The authors achieved increased stability with the double coating, and release studies demonstrated pH responsiveness, protecting insulin from degradation in the gastric environment.

In vivo studies are essential to evaluate the performance of formulations in biological systems. In this regard Moreira et al. [193] orally administered PCL nanoparticles loaded with α-tocopherol to rats with polyarthritis, a model equivalent to rheumatoid arthritis. They compared the results with free and intraperitoneally administered α-tocopherol. The nanoencapsulated α-tocopherol demonstrated greater efficacy, improving joint inflammation and systemic oxidative stress in the rats.

Modifications to PCL, considering the purpose of nanoformulation, can be beneficial as introducing functional groups into the polymer structure adjusts its hydrophilicity, biodegradation rate, and bioadhesion. PCL functionalization allows modifications by incorporating drugs, bioactive fractions, and stimuli-responsive components. Generally, functionalizations are performed on ε-caprolactone monomers through a bond due to its simplicity and minimal interference with ROP. Water-soluble polymers such as PEG (Figure 5c), mPEG, polyethylene oxide (PEO), and polyethyleneimine (PEI) are copolymerized, while hydrophilic polymers like starch and clay are blended with PCL [234–235].

PEGylation of PCL, as with PLGA, enhances the molecule’s hydrophilicity and prevents absorption by the reticuloendothelial system, thereby increasing blood circulation time. Additionally, it impacts the crystallinity of the polymer by lowering the degradation temperature, shortening the crystallization time, and increasing the average crystal size [236].

In this context, PEG-PCL has been used as a carrier for hydrophobic drugs. Kurd et al. [189], utilized this copolymer to encapsulate indinavir, an antiretroviral with limited oral application due to its biopharmaceutical properties. Pharmacokinetic studies in their article demonstrated increased plasma concentrations of indinavir administered as a mPEG-PCL nanoparticle, along with prolonged systemic circulation compared to the free drug.

Poudel et al. [195] reported promising results using PEG-PCL for the nanoencapsulation of nucleic acids, addressing the primary challenge of gastric protection in oral administration. The nucleic acids formed complexes with PEI, a cationic polymer, and were encapsulated within PEG-PCL nanoparticles to prevent degradation. The nanocarrier protected the complexes in a simulated gastric fluid environment, releasing them in the intestinal fluid due to the action of lipases. Oral administration of these systems in mice showed the presence of the complex in the intestine and its delivery to the colon, successfully inducing gene expression following absorption.

Modifications to PCL have shown potential in optimizing NPs. The studies analyzed in this review highlight advantages such as stability, controlled release, increased systemic absorption, improved hydrophilicity, and protection of drugs sensitive to the gastric environment, preventing early degradation The combination of these benefits, demonstrated in animal models, positions PCL as a viable nanocarrier option for the oral administration of drugs.

**4.2.3 Eudragit^®^.** Eudragit^®^ (Figure 5d) is a line of methacrylate-based polymers registered by GmbH & Co. KG Darmstadt in Germany, first marketed in the 1950s. Its chemical name is poly(butyl methacrylate-*co*-(2-dimethylamino) ethyl methacrylate-*co*-methyl methacrylate), and it belongs to the class of polymethacrylates, which are cationic or anionic synthetic polymers of dimethyl aminoethyl methacrylate, methacrylic acid, and methacrylic acid esters in varying proportions. These polymers are synthesized through the polymerization of acrylic and methacrylic acids or their esters [168].

Classified as cationic, anionic, and neutral, Eudragit^®^ is available in the form of powders, granules, aqueous dispersions, and organic solutions. The most commonly used types for oral administration in NPs are Eudragit^®^ E, Eudragit^®^ RL, Eudragit^®^ RS, and Eudragit^®^ S100 [237]. Table 4 describes studies using Eudragit^®^-based NPs.

Eudragit^®^ E is a cationic copolymer that is soluble at gastric pH due to the hydration of dimethylamino groups, which undergo protonation under these conditions. This property makes it suitable for oral administration formulations [238]. Chaurasia et al. [179] developed NPs of Eudragit^®^ E100 loaded with naringenin to enhance its oral bioavailability and anticancer potential. Cancer cells are negatively charged; therefore, through electrostatic attraction, the drug delivery from NPs with a positive surface charge was passively directed, enhancing the therapeutic effect. The anticancer activity was evaluated in mice, showing a 96-fold increase in bioavailability and a 16-fold increase in cytotoxicity, with significant tumor suppression. The cationic properties of the NPs improved the efficacy of naringenin against colorectal cancer.

The ammonium groups present in the Eudragit^®^ RL molecule are responsible for its permeability. This cationic polymer is composed of methyl methacrylate, ethyl acrylate, and a smaller proportion of methacrylic acid ester with quaternary ammonium groups. Its structure makes it highly stable and hydrophobic, with excellent extrudability and swelling properties that are independent of pH [239–240].

Considering the gastrointestinal stability of Eudragit^®^ RL, Pawar et al. [181] utilized the polymer to develop NPs for stabilizing Pickering nanoemulsions for oral administration. The formulation does not contain any surfactants; the nanoparticle itself acts as the stabilizer. Ketoprofen is encapsulated within the Pickering system, where the Eudragit^®^ particles protect the drug and enable controlled release based on the pH of the gastrointestinal tract. In vitro studies demonstrated prolonged ketoprofen release for up to 6 h (82%), whereas the control formulation without NPs completed the release process within 1 h. These findings highlight the polymer’s pH sensitivity as a critical factor in achieving controlled release.

**Figure 6:**
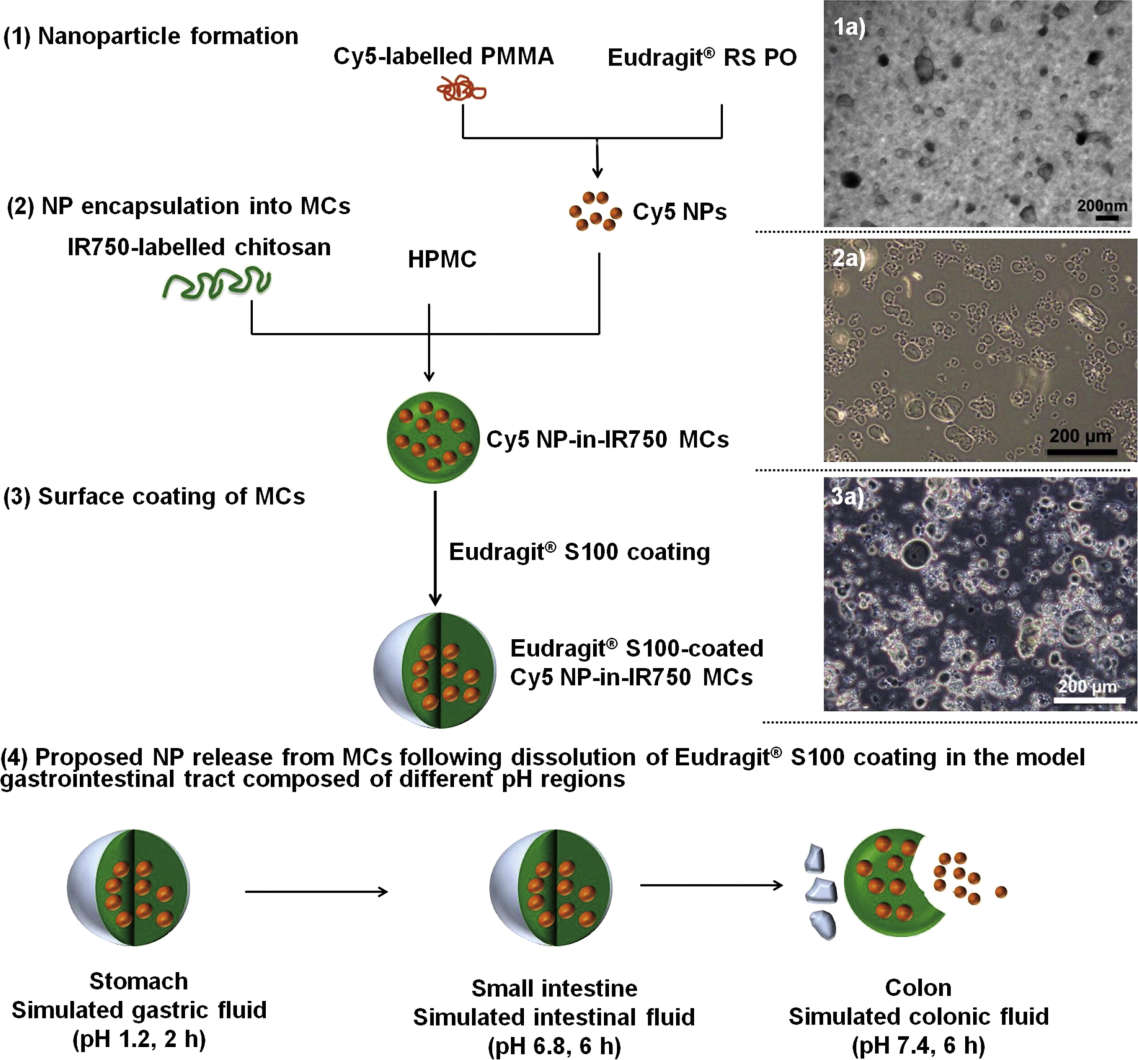
Preparation of nanoparticle-loaded microcapsule systems tracked by multispectral optical imaging. (1a) Transmission electron microscopy (TEM) image of nanoparticles (scale bar 200 nm); (2a) optical micrograph of microcapsules with no coating (scale bar 200 μm); (3a) optical micrograph of microcapsules after coating with Eudragit S100 (scale bar 200 μm). (4) Schematic of the digestive tract (https://www.sciencedirect.com/topics/agricultural-and-biological-sciences/digestive-tract) showing the various pH regions and residence times that must be considered in the design of carrier systems. Figure 6 was reprinted from [182], European Journal of Pharmaceutics and Biopharmaceutics, vol. 94, by Y. Ma; A. V. Fuchs; N. R. B. Boase; B. E. Rolfe; A. G.A. Coombes; K. J. Thurecht, “The in vivo fate of nanoparticles and nanoparticle-loaded microcapsules after oral administration in mice: Evaluation of their potential for colon-specific delivery”, pages 393-403, Copyright (2015), with permission from Elsevier. This content is not subject to CC BY 4.0.

Eudragit^®^ RS shares the same characteristics as RL but differs in the proportion of ammonium groups, containing only 5% quaternary ammonium. This composition significantly reduces its intestinal permeability [241]. The use of Eudragit^®^ RS in combination with other materials can compensate for this limitation. For instance, Ma et al. [182] compared the tissue distribution of free Eudragit^®^ RS NPs and chitosan microcapsules loaded with enteric-coated NPs after oral administration in mice (Figure 6). The microcapsules enhanced the delivery of NPs to the colon, with complete release observed in the lower part of the small intestine. This suggests that the protection provided by the nanoparticle was effective.

The anionic polymers Eudragit^®^ S100, Eudragit^®^ L100, and Eudragit^®^ L100-55 are composed of poly(methacrylic acid-*co*-acrylates). Eudragit^®^ L100 distinguishes itself from S100 by having a higher proportion of active carboxylic groups (48.3%), which contributes to their pH-dependent solubility profiles. Eudragit^®^ L100 dissolves at pH values above 6, while S100 dissolves at pH values above 7. Eudragit^®^ L100-55, in contrast, is a methacrylic acid/ethyl acrylate copolymer that dissolves at pH values greater than 5.5 [242].

Zhang et al. [178] produced NPs of thiolated Eudragit^®^ L100 (Eul-cys) and reduced glutathione (GSH) loaded with insulin. Thiomers are mucoadhesive polymers capable of covalently binding to mucus. The thiolated NPs exhibited a high degree of swelling in the intestinal region and demonstrated rapid in vitro release of insulin. The authors explained that the thiolated complex swelled significantly at intestinal pH, interacting with the mucus and mucin to form disulfide bonds with the sulfhydryl groups. This interaction created a physical and mechanical barrier that facilitated nanoparticle transport. The study concluded that combining these polymers represents a promising strategy for increasing the oral bioavailability of insulin. Furthermore, studies like this contribute to advancing the understanding of Eudragit as an oral nanocarrier.

**4.2.4 Alternative synthetic polymers.** New polymers with the versatility to enable the oral delivery of molecules with limited solubility are emerging, as described in Table 4. Polyanhydride NPs are a notable example of this innovation, offering biocompatibility, biodegradability, and the ability to promote sustained drug release.

One of the most widespread examples within this group is the poly(methyl vinyl ether-*co*-maleic anhydride) (Figure 5e) derivatives, marketed as Gantrez^®^. This polymer is an alternating copolymer of methyl vinyl ether and maleic anhydride. Its primary characteristic is its ability to establish bioadhesive interactions in the GIT due to the formation of carboxylic groups during the hydrolysis of anhydride residues in the copolymer. These carboxylic groups form hydrogen bonds with mucosal constituents, enhancing adhesion. Another advantageous feature of this polymer is the ease of modifying its NPs to improve their physicochemical and biological properties, making it a versatile carrier for drug delivery applications [169,243].

The mucoadhesion capacity of Gantrez^®^, as demonstrated by Patel et al. [186] was responsible for the higher bioavailability of its NPs loaded with rifampicin compared to another nanosystem using poly(ethylene sebacate), a hydrophobic polymer. The study compared lung accumulation after the oral administration of rifampicin-loaded NPs produced with these two polymers. Rifampicin, a tuberculostatic drug, exhibited improved targeting and retention in the lungs when delivered using Gantrez^®^ NPs due to their enhanced mucoadhesive properties.

Coating nanoparticles with PEG is a well-established strategy in oral drug delivery systems, as discussed in this review. Thus, Inchaurraga et al. [185] studied the effect of pegylation on Gantrez^®^ NPs mucus permeability using PEG of varying molecular weights (PEG 2000, PEG 6000, and PEG 10000). Although the uncoated NPs showed mucoadhesive properties in the stomach, those with PEG reached the epithelial surface of the intestine, and the highest permeations occurred with PEG of a lower molecular weight. The study did, however, show a coating limit for efficiency in mucus permeation. According to the authors, high PEG density makes it difficult for the NPs to diffuse, as the coating is less flexible due to entanglements between the macrogol chains (which facilitates interaction with the mucus). This decreases their ability to reach enterocytes, which is why the entanglement of PEG chains was suggested as a factor that negatively influences bioadhesion.

Other studies are being carried out with polyanhydro NPs, demonstrating their versatility for conjugation with various substances with promising results. Iglesias et al. [184] compared various ligands and demonstrated that the use of mannosamine consistently increases the permeative capacity through mucus. Inchaurraga et al. [183] investigated another type of coating with characteristics similar to PEG, that is, thiamine. In this study, different coating methods, such as post-synthetic adsorption and desolvation, were applied to nanoparticles, and both approaches yielded comparable physicochemical and biodistribution properties. The strategy aimed to enhance gastrointestinal transit and epithelial interaction. This opens a discussion regarding the selection of less costly methods with greater potential for industrial scalability.

Lipophilic drugs have limitations in being loaded into poly(methylvinylether-*co*-maleic anhydride). NPs, due to the characteristics of the polymer itself, so modifications using cyclodextrins have been used to improve this performance. The increase in oral bioavailability with this strategy occurred with atovaquone [244], and camptothecin [245]. Lucio et al. [215] demonstrated the antidiabetic effect of glibencamide using this approach. However, Huarte et al. [246] comparing Gantrez^®^ NPs with different conjugates (PEG and beta-cyclodextrin), demonstrated that although both increased the relative oral bioavailability of camptothecin, this value was 2.6 times higher with PEG.

The properties of PEG, which were discussed in this review, justify this result. However, the incorporation of cyclodextrin facilitates the encapsulation of lipophilic drugs, leading to increased solubility, oral bioavailability, and stability. In addition, it modulates and sustains the release of molecules from PNs through their interactions, forming an inclusion complex with reversible bonds [247]. Therefore, they are rapidly hydrolyzed because they are not metabolized by gastric acids or amylase [248].

Modifications using PEG are a common approach in polymeric nanocarriers for oral administration. Chaturvedi et al. [214] fabricated PHB NPs conjugated with PEG and deoxycholic acid (DOCA), loaded with insulin. This system was granulated and coated with Eudragit^®^, which prevented the release of insulin in the acidic environment with 11.6% bioavailability. There was rapid cellular uptake and internalization, with increased intestinal absorption and a hypoglycemic effect sustained for 24 h after oral administration.

PHB (Figure 5f) is a biodegradable and promising polymer that, upon degradation, generates 3-hydroxybutyric acid, a component produced by endogenous ketogenesis, suggesting its biocompatibility. Its degradation is slow and mediated by enzymatic hydrolysis, starting with the diffusion of water molecules in the polymer matrix, cleaving its chains. Lipases and esterases are responsible for the erosion of the polymer surface [249].

Niu et al. [211] developed a novel oral peptide delivery nanosystem using insulin as a model. It consisted of an insulin complex with a cell-penetrating peptide (CPP) modified hydrophobically with lauric acid (C12) and octa-arginine (r8). The entire complex was protected by a coating layer of PEGylated polyamino acids (PGA-PEG) to prevent nanoparticle aggregation and enhance mucodiffusion. The nanosystem was stable, protected insulin from degradation in the gastrointestinal tract, diffused through intestinal mucus, and achieved an uptake of 47.59 ± 5.79%. In vivo studies demonstrated that retention in enterocytes did not translate into enhanced systemic absorption.

Ha-Lien Tran et al. [187] explored complex interpolymer structures with HPMC and chitosan linked with TPGS (polyethylene glycol ᴅ-α-tocopherol succinate) for the oral administration of docetaxel. Two nanoparticle manufacturing strategies were carried out: In the first, HPMC-chitosan was formed, conjugated with TPGS, and followed by docetaxel loading. In the second, the drug was first loaded with TPGS and then linked to the HPMC-chitosan conjugate. The first production method avoided release at the beginning of the GIT. Additionally, the chitosan amino group involved in the conjugate binding made it impossible to dissolve this biopolymer at acidic pH. PVA acted not only as a stabilizer but also as a cross-linker. There was greater permeability in CaCo_2_ cells and the cellular uptake, demonstrated facilitated transport of NPs with docetaxel from the small intestine to the bloodstream for subsequent targeting to the tumor site.

Considering that spherical NPs are most studied for oral administration Banerjee et al. [213] manufactured polystyrene particles with different sizes and shapes to analyze the uptake and transport through intestinal cells, with emphasis on the rod shape compared to spheres or discs. Although the rod shape improved the efficacy of NPs for oral administration, in vivo studies are necessary to elucidate and confirm the results found.

The alternative synthetic polymers discussed in this section provide promising solutions to address the challenges of oral drug delivery. Their key attributes include mucoadhesion, controlled release, ease of structural modification, and the capacity to encapsulate hydrophobic molecules. Nevertheless, additional in vivo studies are essential to confirm the efficacy and safety of these systems and assess their potential for industrial scalability.

#### 4.3 Hybrid nanoparticles – the future in oral drug delivery

Solid lipid nanoparticles (SLNs) possess a crystalline lipid core stabilized by interfacial surfactants, which can encapsulate lipophilic molecules due to the intrinsic properties of lipids. Hydrophilic molecules, in turn, tend to localize at the surface or within interfacial regions, depending on the nanoparticle composition and the nature of the surfactants used. These SLNs are effective as delivery systems for drugs, nucleic acids, and vaccines, offering advantages such as high drug entrapment efficiency, ease of production, biocompatibility, scalability, and targeted delivery. However, they also present limitations, including reduced stability, rapid release of the active ingredient, excessive polydispersity, and a low weight-to-volume ratio, which restricts the payload that can be effectively encapsulated [250].

**Figure 7:**
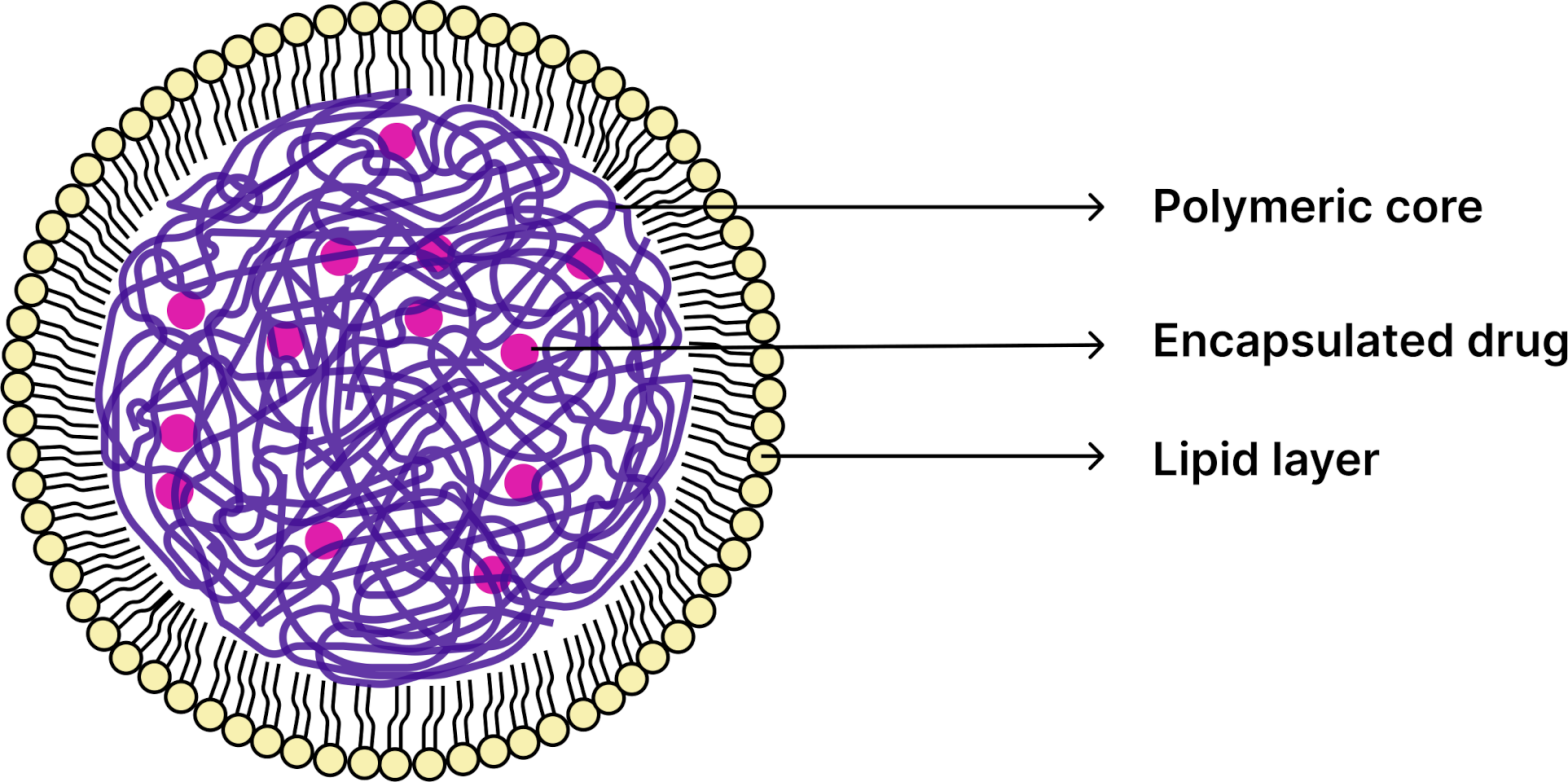
General structure of a lipid–polymer hybrid nanoparticle. The core consists of polymer chains and encapsulated drug. The external lipid portion can be conjugated with different constituents such as PEG, antibodies, folates, and others, according to the therapeutic target. Created with Figma.com (https://figma.com/).

In this context, a strategy involving lipid–polymer hybrid nanoparticles (LPHNs) to leverage the advantages of biodegradable PNs and biomimetic phospholipids has been investigated to overcome the limitations of individual systems. These hybrid NPs (Figure 7) are formed from a polymer core wrapped in a lipid layer. They feature a drug-encapsulating polymer core, a lipid layer surrounding the polymer core, and an external lipid–PEG layer in some cases. For oral administration formulations, this type of nanocarrier offers gastrointestinal stability, which is a significant barrier for oral nanostructured systems [251].

The inner lipid membrane may play a critical role in drug protection, acting as a hydrophobic barrier that reduces water diffusion and minimizes chemical degradation processes within the NP. The outer lipid layer may modulate the NP’s interactions with the immune system, influencing plasma protein adsorption and, consequently, affecting systemic circulation time. [252]. This is because PNs, due to their hydrophobic surface, are recognized as foreign material, triggering an immune response, short circulation time, and rapid elimination [251].

Table 5 illustrates research with hybrid NPs for oral administration, aimed at increasing encapsulation efficiency for therapeutic molecules such as peptides. Boushara et al. [253] incorporated 2% methocel and doubled the encapsulation efficiency of insulin compared to formulations with conventional lipid nanocarriers, protecting it from degradation in the gastrointestinal tract. The hydrophobic nature of lipid nanoparticles impedes the encapsulation of hydrophilic molecules like insulin. Methocel is a non-ionic, hydrophilic polymer that is stable from pH 2 to 13, acting as a surfactant, stabilizing the emulsion and reducing surface and interfacial tension.

**Table 5:** Research using hybrid nanoparticles for oral use.

Polymer	Lipid base	Drug/BCS	Therapeutic class	Preparation technique	Ref.
Objective Results (Release, In vitro test, In vivo test)					

Dextran	Pesirol; bovine serum albumin	astaxanthin (II)	antioxidant	Emulsification gelatio	[254]
Objective: Develop polymer-lipid hybrid NPs with gastrointestinal stability. Release: 50% of the nanoencapsulated drug was released in 6 h versus 80% of the free drug. In vitro test: Antioxidant activity at very low concentrations (0.25 μg/mL). In vivo test: –					

Methocel PLGA	trimyristin soy lecithin	insulin (N/A^a^)	hypoglycemic agent	emulsification, solvent evaporation	[253]
Objective: Investigate the feasibility of hybrid nanoformulations with a focus on encapsulation efficiency. Release: – In vitro test: Low toxicity and 60% cell viability. In vivo test: –					

PEG PLGA	trimyristin soy lecithin	insulin (N/A^a^)	hypoglycemic agent	emulsification, solvent evaporation	[255]
Objective: Investigate and characterize hybrid NPs demonstrating potential for oral insulin administration with an emphasis on increasing encapsulation stability. Release: – In vitro test: 75% cell viability. In vivo test: Oral free insulin had no effect, while LPHNs had a longer and stronger hypoglycemic effect compared with subcutaneous insulin.					

PEG-PCL	geleol mono diglycerides nf	larotaxel (IV)	antineoplastic	dialysis	[256]
Objective: Investigate the role of nanoparticle stability in their performance for oral drug delivery. Release: – In vitro test: Low cytotoxicity in Caco-2 cells. Transcytosis determined that the intracellular larotaxel content is 1.65 times higher in NPs. In vivo test: LPHN showed an area under the curve 5.38 times higher when compared to the orally administered drug.					

PLGA	phosphatidylcholine	bromelain (N/A^a^)	anti-inflammatory antineoplastic	emulsification, solvent evaporation	[257]
Objective: Develop a lipid-polymeric formulation encapsulating bromelain to increase its stability at acidic pH and efficiency in oral delivery. Release: After 5 days, the release of bromelain from PLGA-PC NPs was 34% at pH 1.2 and 20.36% at pH 6.8, compared to 30.1% and 18.8% from PLGA NPs. In vitro test: Cell viability >80% and 98.4% cumulative transport across the intestinal barrier after 4 h in the cellular uptake study. In vivo test: –					

PLGA	dimethyldioctadecyl- ammonium (DDAB)	magnolol (N/A^a^)	anti-inflammatory	emulsification	[258]
Objective: Prepare a hybrid nanoparticle system for oral administration of magnolol to combat ulcerative colitis. Release: Free magnolol demonstrated faster release compared to NPs after 6 h. Magnolol encapsulated in PLGA exhibited a slow and sustained release over a period of 7 days. In vitro test: Internalization by macrophages through CD44-mediated endocytosis. Increased levels of inflammatory cytokines such as NO, IL-6, and TNF-α. In vivo test: Increased tight junction protein expression and protection of the intestinal mucosa, without histopathological damage in treated mice.					

PLGA, chitosan	1,2-distearoyl-sn-glycero-3-phosphoethanol-amine-*N*- methoxy (polyethylene glycol)-2000; LIPOIDE S100	curcumin (IV)	antineoplastic	nanoprecipitation	[259]
Objective: Obtain rapid mucus permeation, enhanced cellular uptake and transepithelial absorption through hydrophilic polymers and chitosan conjugated with vitamin B12. Release: – In vitro test: Improved diffusion through mucus and improved endocytosis in CaCo-2 cells increasing cellular uptake. In vivo test: The *T*_max_ obtained for the LPHN was 7.98 times higher compared to the curcumin suspension.					

PLGA, chitosan	1,2-distearoyl-*sn*-glycero-3-phosphoethanol-amine-*N*- methoxy (polyethylene glycol)-2000; LIPOIDE S100	curcumin (IV)	antineoplastic	nanoprecipitation	[259]
Objective: Obtain rapid mucus permeation, enhanced cellular uptake and transepithelial absorption through hydrophilic polymers and chitosan conjugated with vitamin B12. Release: – In vitro test: Improved diffusion through mucus and improved endocytosis in CaCo-2 cells increasing cellular uptake. In vivo test: The *T*_max_ obtained for the LPHN was 7.98 times higher when compared to the curcumin suspension.					

Chitosan	cardamom oil, PEG laurate	felodipine (II)	antihypertensive	microwave emulsification	[260]
Objective: Prepare and characterize oral hybrid nanocarriers to increase solubility, bioavailability and control delivery to increase patient compliance. Release: Sustained release over 36 h of the hybrid NPs and the pure drug was released rapidly. In vitro test: – In vivo test: Drug suspension showed lower permeation than felodipine NPHLs.					

^a^N/A: Refers to substances to which the biopharmaceutical classification cannot be applied because they are biological or similar molecules.

Wang et al. [254] fabricated a stable hybrid nanoparticle in the gastrointestinal tract through in situ conjugation between oxidized dextran and bovine serum albumin. This was done to encapsulate astaxanthin, a lipophilic compound, which had its antioxidant activity increased in aqueous environment and sustained release in simulated gastrointestinal fluids. Oxidized dextran has aldehyde groups that stabilize glyceryl distearate, and through in situ conjugation, form the hybrid NPs. Stability was achieved due to the covalent bond between the oxidized dextran aldehyde and the amino group of bovine serum albumin. The results demonstrate the feasibility of encapsulating lipophilic molecules through this delivery system for oral administration.

Oral administration of insulin is a necessity for users due to dosage convenience, causing nanocarrier systems to be studied exhaustively aiming at various tools to increase the entrapment of this molecule. Boushra et al. [255] incorporated two polymers into SLNs, resulting in bi-polymeric lipid nanocarriers. PEG was added to the internal aqueous phase and PLGA to the lipid phase, achieving 50% (20% without the bi-polymer) encapsulation efficiency and preserving the chemical stability and biological activity of insulin.

Promising results with LPHNs may represent an alternative for molecules with low oral bioavailability, which is why Drais and Hussein [260] prepared hybrid nanocarriers loaded with felodipine to increase their solubility and improve their therapeutic effect. The use of chitosan in this formulation demonstrates the wide applicability of this biopolymer as a nanocarrier. The release and intestinal permeation studies demonstrated prolonged release and stability at the pH of the gastrointestinal tract. In addition, encapsulation efficiency was above 70%.

The use of combined compounds and vehicles has been frequently used in the development of nanocarriers, especially for oral drug administration. The gastrointestinal tract is an important physiological barrier with enzymes, changes in the chemical environment, and long path; hence, the encapsulation of hydrophilic and hydrophobic molecules has limitations. Therefore, lipid–polymer hybrid NPs represent the future of nanocarriers, as their polymeric core wrapped in a lipid layer [261–262] has demonstrated satisfactory results for controlled drug release, increased encapsulation rates, and stability in the GIT. These characteristics represent an advance in the development of NPs for oral administration.

## Conclusion

This review reinforces the pivotal role of polymers in advancing oral drug delivery through nanoparticulate systems. The structural and functional diversity of natural and synthetic polymers enables the development of nanocarriers capable of protecting drugs from degradation, improving solubility, modulating release profiles, and enhancing bioavailability. Natural polymers, such as chitosan and alginate, provide advantages in terms of biocompatibility, mucoadhesion, and permeability, while synthetic polymers like PLGA and PCL offer better control over degradation rates and greater flexibility for chemical modification and are also biocompatible. Hybrid systems combining different polymer classes and lipids have demonstrated synergistic effects, although they still face production and scalability challenges.

In this context, the lack of standardization in nanoparticle characterization, poorly defined absorption mechanisms, and the scarcity of in vivo studies with proven clinical efficacy remain significant limitations in the development of oral polymeric nanoformulations. Another barrier to the advancement of clinical application is the absence of clear regulations for this type of formulation.

Smart polymers with the ability to release drugs in response to specific stimuli represent a tangible future direction in this field, aiming to optimize therapeutic profiles. Furthermore, ensuring the safe administration of oral polymeric nanocarriers will require scalable manufacturing technologies supported by bioinformatics and artificial intelligence. Therefore, advances in polymer science and nanotechnology are essential for expanding the application of these systems to peptides, nucleic acids, genes, and poorly soluble molecules. All of this consolidates PNs as a promising strategy for more efficient oral therapies.

## Data Availability

Data sharing is not applicable as no new data was generated or analyzed in this study.
